# Investing in human development and building state resilience in fragile contexts: A case study of early nutrition investments in Burkina Faso

**DOI:** 10.1371/journal.pgph.0001737

**Published:** 2023-03-29

**Authors:** Chris Desmond, Kathryn Watt, Palwendé R. Boua, Candice Moore, Agnes Erzse, Hermann Sorgho, Karen Hofman, Toussaint Roumba, Halidou Tinto, Kate A. Ward, the INPreP group

**Affiliations:** 1 SAMRC/ Wits Centre for Health Economics and Decision Science, PRICELESS SA, Faculty of Health Sciences, University of Witwatersrand School of Public Health, Johannesburg, South Africa; 2 Centre for Rural Health, School of Nursing and Public Health, University of KwaZulu-Natal, Durban, KwaZulu-Natal, South Africa; 3 Clinical Research Unit of Nanoro (CRUN), Institut de Recherche en Sciences de la Santé, Nanoro, Burkina Faso; 4 Sydney Brenner Institute for Molecular Biosciences (SBIMB), University of Witwatersrand, Johannesburg, South Africa; 5 Department of International Relations, School of Social Sciences, Faculty of Humanities, University of the Witwatersrand, Johannesburg, South Africa; 6 SAMRC Developmental Pathways for Health Research Unit, School of Clinical Medicine, University of the Witwatersrand, Johannesburg, South Africa; 7 Global Health Research Institute, School of Human Development and Health, Faculty of Medicine, University of Southampton, United Kingdom; 8 Medical Research Council Lifecourse Epidemiology Centre, University of Southampton, Southampton, United Kingdom; African Population and Health Research Center, KENYA

## Abstract

Maternal and early malnutrition have negative health and developmental impacts over the life-course. Consequently, early nutrition support can provide significant benefits into later life, provided the later life contexts allow. This study examines the limits of siloed investments in nutrition and illustrates how ignoring life-course contextual constraints limits human development benefits and exacerbates inequality, particularly in fragile contexts. This case study focuses on Burkina Faso, a country with high rates of early malnutrition and a fragile state. We modelled the impact of scaling up 10 nutrition interventions to 80% coverage for a single year cohort on stunting, nationally and sub-nationally, using the Lives Saved Tool (LiST), and the consequent impact on earnings, without and with a complementary cash-transfer in later life. The impact on earnings was modelled utilising the well-established pathway between early nutrition, years of completed schooling and, consequent adult earnings. Productivity returns were estimated as the present value of increased income over individuals’ working lives, then compared to estimates of the present value of providing the cost of nutrition interventions and cash-transfers. The cost benefit ratio at the national level for scaled nutrition alone is 1:1. Sub-nationally the worst-off region yields the lowest ratio < 0.2 for every dollar spent. The combination of nutrition and cash-transfers national cost benefit is 1:12, still with regional variation but with great improvement in the poorest region. This study shows that early nutrition support alone may not be enough to address inequality and may add to state fragility. Taking a life-course perspective when priority-setting in contexts with multiple constraints on development can help to identify interventions that maximizing returns, without worsening inequality.

## Introduction

The consequences of malnutrition prior to conception, during pregnancy and in early childhood, for the individual and their caregivers include not only immediate suffering and health risks, but long-term health and developmental challenges, including heightened risk of untimely death [1, 2]. Given the long-term consequences, interventions to avoid or mitigate malnutrition have the potential to lead to significant benefits over the life-course [3]. However, such benefits accrue only to the extent the context in subsequent life stages allows. We outline how, when investing in early nutrition to improve health and human development outcomes, a failure to recognise and act to improve these life-course interactions through later life inputs can miss opportunities to realise the full benefits of nutrition interventions.

We focus on Burkina Faso, a country with high rates of early malnutrition which threatens human development. Prioritising the improvement of these early life nutrition outcomes is critically important. However, we show that doing so in a context characterised by multiple adversities, high rates of inequality and state fragility may lead to limited and uneven benefits. As a consequence, nutrition interventions may reinforce or magnifying unequal outcomes, which in the context of state fragility partially caused by such inequality may even lead to harm. With this in mind, we examine the possibility of mitigating this risk, taking a life course perspective. by using a novel human development framework. The framework distinguishes between factors which protect human development potential, those which contribute to the realization of this potential, and those which facilitate its utilization. These factors occur at every life stage but are concentrated in early childhood, adolescence, and adulthood respectively [4]. Interventions in early-childhood, including nutrition interventions, protect human development potential [5, 6]. They also contribute to the realization of this potential, but full realization requires a conducive environment throughout childhood and adolescence, such as school attendance [7]. Realized potential requires an appropriate environment for its utilization; without it, the realized human development cannot be put to full use. Our framework can be used to understand why promising early intervention outcomes can fade in later life for those living in challenging contexts [8].

Within such a human development framework, the value of interventions to improve early nutrition is determined by a person’s environment across their life course. A narrow focus on nutrition, be it nutrition-specific of nutrition sensitive may miss that in contexts characterized by high levels of inequality, a given improvement in nutrition outcomes will have a very different value for children at opposite ends of the distribution [4]. The outcome will have greater value for children at the upper end given they will have more opportunities than children at the lower end to realize and utilize the potential protected by the intervention. While a given improvement in a nutrition outcome will have a smaller value for children at the lower end of the distribution, the frequency that children at the lower end are likely to experience that improvement following an intervention will typically be greater, because they suffer much more from malnutrition. As a result, following intervention, each child that benefits will benefit less than a child from the upper end of the distribution, but more children in the lower end will benefit because the need is greater. At the population level, the value of a given improvement and the frequency/magnitude of improvement, therefore, have opposite impacts on inequality, requiring an examination of which effect is likely to dominate. In contexts such as Burkina Faso, where malnutrition affects almost the entire distribution, this latter influence may not be enough to counter the influence of unequal life chances. This should not be taken to suggest that it is more worthwhile protecting the potential of those who are likely to already have opportunities to realize and utilize that potential, but rather that we should not stop at protection when there are known barriers to realization and utilization.

In this paper we examine the potential benefits and limits of investing in the scale up of 10 evidence-based maternal and child nutrition-specific interventions to prevent and mitigate the consequences of malnutrition in Burkina Faso, with and without a later-life cash-transfer intervention to mitigate school drop-out. This case study allows us to investigate the limitations of early nutrition support as a transformative instrument in face of state fragility. Further it allows us to explore how these limitations can be addressed by taking a life-course perspective when identifying priority interventions. It also highlights the bi-directional interactions between efforts to improve human development and those to build a more resilient state.

### Human development and state resilience

Our focus on human development over the life-course, rather than narrow nutrition, health or development outcomes within one life stage, draws attention to the role of the state. Non-state actors can step in to address single outcomes, but it is not as easy for them to take on sets of interventions which span across life stages. Consequently, the ability of the state to identify and implement these sets of interventions is arguably critical to maximizing returns, especially when there are multiple constraints on development. The challenge is that in contexts characterized by multiple constraints, the state often has limited ability to identify and implement the necessary interventions, most notably in fragile states. In such situations, donors may consider bypassing the state, and focusing on those outcomes they can improve. But bypassing the state in fragile contexts misses an opportunity to improve the ability of the state to offer services, while potentially contributing to legitimacy challenges. Human development investments may be key to addressing fragility, just as the state is key to maximizing human development outcomes. We suggest that it is best to consider the bi-directional relationship between state fragility and human development outcomes by asking how investments in addressing one can act to improving outcomes for the other. There is increasing recognition in the literature on addressing state fragility of the importance of considering health, education and other human development investments and outcomes. What is missing is a consideration of state fragility in the literature on prioritizing among health and human development investments.

There is a gradual shift among donors and international partners in thinking about addressing fragility from state building to building state resilience [9], including through investments in human development, as a powerful means of preventing conflict and building capacity to respond to future crises [10]. We understand building state resilience as measures taken to protect the political, economic, and social environments of the state from crises and shocks. Focusing on resilience is substantially different from the previous obsession with state building which was characterized by efforts to determine “the right sequence or combination of state-building reforms” [9]. No longer as preoccupied with institution building, global development actors, including the World Bank and United Nations, are embracing the view that “structural reforms towards positive resilience” are the best means of preventing conflict and other crises [10].

Underpinning this shift is an understanding that “resilience through investment in inclusive and sustainable development—including addressing inequalities, strengthening institutions and ensuring that development strategies are risk-informed is the best means of prevention” [11]. A key aspect of sustainable development is ensuring human development outcomes improve. Recognizing this, the OECD, in its forthcoming States of Fragility Report (2022) will include a human capital dimension in its fragility framework [12]. Including this human dimension allows for measurement of risks affecting human development, monitoring levels of health, education, socio-economic vulnerability and inequality, as well as the presence of institutions and services and infrastructure to mitigate risks [13].

This growing recognition of the role of investments in human capital, or as we prefer, human development, in addressing the causes and consequences of state fragility, raises the question of how to determine which investments to make, including considering whether such decisions have consequences for efforts to build resilience.

In fragile contexts, human development indicators tend to be poor and therefore any of a wide range of interventions could generate high returns. There are a variety of economic evaluation approaches which can be used to identify the highest priority interventions, such as Cost Benefit Analysis (CBA) and Cost Effectiveness Analysis (CEA), but they need to be used with caution in such difficult environments. The challenge with CBA highlighted by our framework is that returns tend to be correlated with how supportive the environment is. Moreover, the implementation costs are likely to be higher, given weakened infrastructure, when delivering services to those who are worst off. This pattern of costs and benefits will lead to interventions for better off populations being prioritized. If these interventions are then implemented, they will reinforce existing patterns of inequality which may undermine efforts to build a resilient state. This can be addressed by incorporating equity as a goal. Having decided on this goal, CEA can be used to identify the most efficient mix of interventions to equalize a human development outcome of interest. But what our framework highlights is that a given outcome differs in value to the individual, depending on the context they will face over their life-course. Therefore, incorporating equity in a single outcome as a goal, while it will help, does not go far enough to prevent interventions prioritized in this way from potentially reinforcing inequalities. Seeking rather to equalize human development opportunities is what is needed. This requires a consideration of the ways in which human development occurs over the life-course and to identify packages of interventions which facilitate this process.

**Fig 1:**
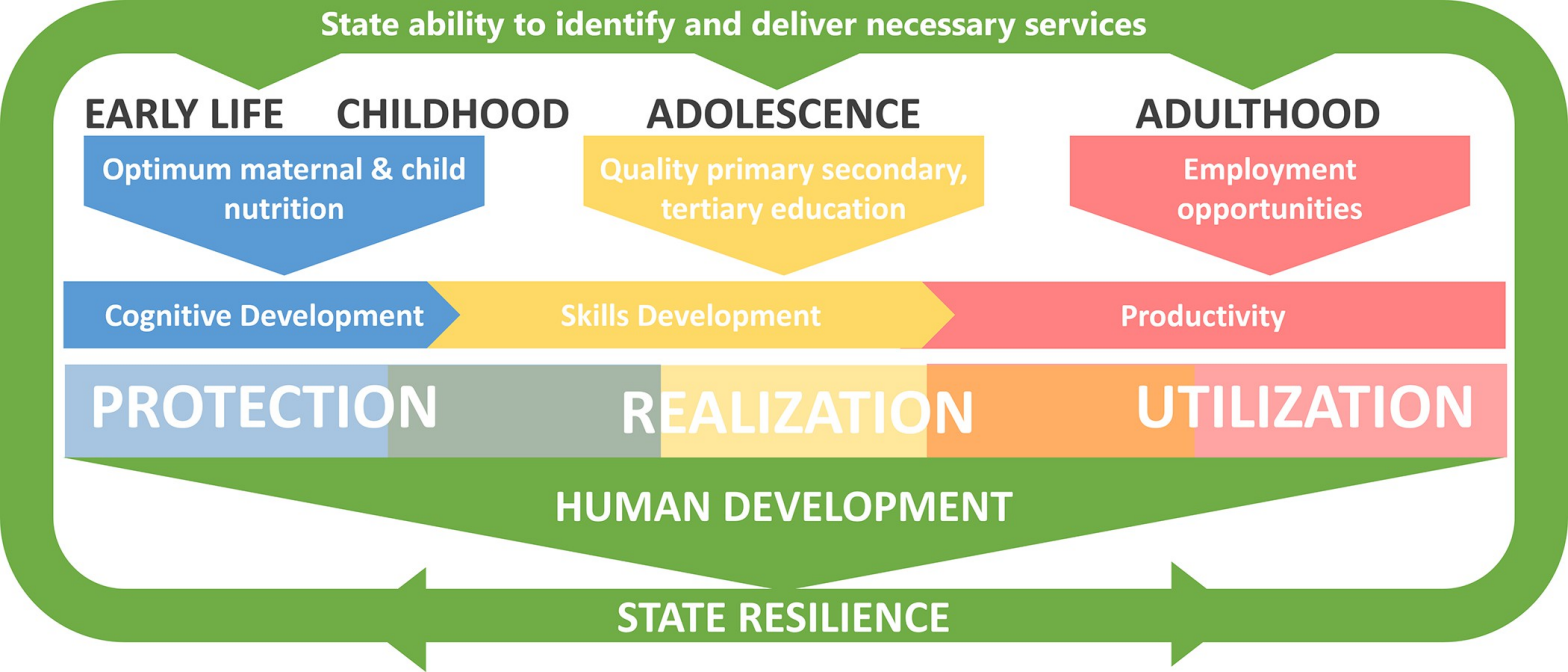
The impact of nutrition interventions through cognitive development, within the human development framework.

Fig 1 summarises our expanded human development framework, including the bi-directional relationship between building state resilience and investing in human development over the life-course. Further, Fig 1 highlights the pathway we examine in our case study: the relationship between investments in early nutrition and human development over the life course, proxied by productivity returns. Maternal and early nutrition interventions protect human development potential through multiple interacting outcomes such as healthy growth, motor and socioemotional development and cognitive development [3]. Consequently, early nutrition intervention via its influence on cognitive development, protects potential, then access to quality education influences the extent to which the potential of such cognitive development will be realized through education, and how opportunities for employment shape the extent to which learnings can be utilized in the labour market. Investments in each of the life stages are influenced by state ability to deliver services, itself a function of state resilience, while improved outcomes influence how resilient the state becomes.

### Case study context

The starting point for our case study is the urgent need to address nutritional challenges in the first 1,000 days of life [3]. We add to this an emphasis on the importance of identifying and addressing constraints on human development over the life-course, and a consideration of how such responses act to help or hinder efforts to build a more resilient state. We take the currently observed constraints on human development across the life stages as an indication of the challenges infants today will face in their future if nothing more is done to address them.

Children and adolescents in Burkina Faso face high rates of poverty, malnutrition and food insecurity, a struggling education system with few progressing past primary school and high rates of child marriage and labour [2, 14, 15]. Burkina Faso is in the bottom 10 countries on the 2020 End of Childhood index, where childhood is most threatened [15]. The country’s highly constrained Human Development, combined with recent intensified security challenges and political instability, bring the country firmly within the realm of fragility [16].

Malnutrition is a particular concern for policymakers in Burkina Faso, with 18% of children under five years underweight, 9% wasted (have low weight-for-height) and 25% stunted (have low height-for-age) [14]. Drivers of malnutrition include low prevalence of exclusive breastfeeding among children aged 0–5 months [14], and poor maternal nutrition, especially among adolescent girls; 62% of women of reproductive age suffer from anemia, and 16% are underweight, factors which carry increased risk of adverse pregnancy outcomes [3, 17–19].

Malnutrition in early life is however only one of many challenges facing Burkinabe throughout childhood and with the transition to adulthood. Approximately 36% of children of primary and secondary school age do not attend school, with girls at greater risk of non-attendance [15, 20]. The quality of education for those in school is poor, with an estimated 86% of 10 year-olds not able to read and understand a simple text by the end of primary school [20]. Lack of pre-school facilities, low levels of teacher training with fewer than 25% of pre-school teachers trained, and poor teacher to student ratio impede school performance [21]. For rural children, constraints also include poor road infrastructure, the absence of an affordable and organized transport system and, as a consequence, long walks to school, and child agricultural labour in rainy seasons [21, 22]. For girls there are additional risks, with over half of adolescent girls (age 15–19) forced into marriage before 18 and one in every 10 girls experiencing pregnancy before the age of 18 [15].

Regional in-country disparities mean that Burkinabe are further constrained if they live in the administrative regions most affected by violence or poverty. In 2019 the Nord region had the highest proportion of individuals considered poor (71%), and 27% of children 0–59 months were stunted (Height/Age z-Score <-2), compared to the Centre region where 5% were poor and 13% of children stunted [14, 23]. The Sahel region has been most badly affected by conflict, with 80% of the schools in the region closed as a consequence [24].

The challenges continue beyond childhood. Developmental opportunities for young adults are constrained by socioeconomic factors including poor employment opportunities outside the agriculture sector [25]. While labour force participation is high, low-productivity agriculture accounts for 80% of employment with two-thirds of workers not being paid [25].

Responding to improve human development indicators, particularly to address regional inequalities, is frustrated by the limited and uneven ability of the state to provide services, a symptom of its fragility. The central government is nearly invisible in the rural areas of Burkina Faso. Tensions have been long in rising, with the federal government standing accused of neglecting its Sahelian regions. The government is perceived as distant and corrupt in these areas, and state agents posted there have done little to change this perception [26]. Conflict has further aggravated an already difficult situation. The January 24, 2022 coup was the result of security concerns of millions of Burkinabe, victims of jihadists, community militias and sometimes regular soldiers [27]. The military announced, following approval by a national conference in February 2022, its intention to preside over a period of transition for 3 years.

More than 1,5 million Burkinabe are displaced (8% of the total population of the country). Twelve percent of the country’s health facilities are currently completely closed, depriving more than 1,9 million people of access to health services [28]. Almost all these facilities closed due to direct attacks by unidentified armed groups. Road networks are poor, hindering access for humanitarian aid [28].

Against this backdrop of multiple adversities and state fragility, this paper examines the potential benefits of interventions to prevent and mitigate early malnutrition in Burkina Faso. We consider not only the returns, but their distribution between regions, given that regional disparities are contributing to social and political instability. Further, we examine how complementary interventions could be used to minimise regional differences and in so doing contribute to efforts to build a more resilient state.

## Materials and methods

### The approach

There is a well-established pathway between early nutrition, years of completed schooling and, consequent adult earnings [29]. We modelled this pathway to estimate the value of productivity returns arising from the roll-out of the identified interventions for a single year birth cohort. Productivity returns, reported as the present value of increased income over individuals’ working lives, are intended to provide a marker of utilized human development, not an estimate of the full value of the intervention. These returns were estimated at national level, and for three of the 3 subnational administrative regions, with and without a selected complementary intervention (a cash transfer) in later life stages. Three regions were selected to represent the differing socio-economic contexts: Centre- the most populous and stable region, Sahel-the region most affected by political instability, and Nord- the poorest region—see Table 1 for a summary of the differences.

**Table 1:** Regional profiles.

Indicator	Centre	Nord	Sahel
Population aged 0–4 years, 2020 [30].	405882	325046	264821
Population density, 2019 [31].	1081,2	106,2	31
Incidence of poverty/ Severity of poverty/ of poverty, 2018 [31].	5,3	70,9	44,2
Household employment income, 2018 (Millions of FCFA) [31].	942998	56754,8	102752
Internally Displaced Persons total, 2022 [32].	1051	212434	575870
Internally displaced Children under 5 years old, 2022 [32].	158	37596	103637
% Children 0–59 months of age (Height/Age z-Score <-2), 2019 [33].	12,5	27,3	43,1
% Children 6–59 months of age (Weight/Height z-score <-2), 2019 [33].	8,5	8,2	15,1

### The interventions

In consultation with local experts, we selected the 10 nutrition-specific interventions for women in the reproductive period and their children 0–24 months which were identified by Bhutta et al: folic acid fortification at reproductive age, multiple micronutrient, calcium and balanced energy protein supplementation in pregnancy, and exclusive breastfeeding and age-appropriate complementary feeding for infants and vitamin A and zinc supplementation for infants, and management of severe acute and moderate acute malnutrition [3].

Several options were considered for inclusion as complementary interventions, including those that improve school access and quality. We settled on cash transfers given the weight of evidence of their effectiveness and the availability of data from Burkina Faso. The Nahouri Cash Transfers Pilot Project was a randomised control trial comparing the impact of conditional vs unconditional cash transfers in rural Burkina Faso on schooling for children 7–15 years of age [34]. Given the varying extent of poverty, and rate of primary and secondary school dropout in each of the administrative regions, see Table 1 and S1 Text, cash transfers may be key in realising the benefits of nutrition interventions. Moreover, they may be particularly important in the worst off regions which currently have very low access to services, which cash transfers could help remedy.

In our model we used an estimate of the effect of the transfer on school dropout based on the outcomes from the conditional transfer arm of the study, which included transfers of US$18 per annum for children 7–10 and US$32 for children 11–15 years of age [34]. Cash transfers, as a nutrition sensitive intervention, would likely have additional impacts on nutrition, these were not modelled. Our focus here was on which complementary interventions would allow a fuller and more equitable realisation of the benefits of early nutrition interventions, rather than generating additional nutrition benefits.

### The models

The Lives Saved Tool (LiST) was used to estimate the effect on stunting (height for age z-score (HAZ)<-2 standard deviations below the median) associated with scaling this package of interventions from current levels of provision (see Table 2) to at least 80% coverage [35]. While Bhutta et al model scale-up of interventions to 90% coverage, we opted to model a lower coverage of 80% as a still ambitious but more attainable scenario, given the challenging context of Burkina Faso [3].

**Table 2:** Key intervention baseline coverage 2022.

Indicator	National	Centre	Nord	Sahel
Folic acid supplementation in pregnancy [LiST default]	14.5	14.5	14.5	14.5
Micronutrient supplementation in pregnancy [39, LiST default]	0	0	0	0
Iron supplementation in pregnancy [33].	67.4	65.9	73.1	45.6
Calcium supplementation in pregnancy [40] [LiST default]	0	0	0	0
Balanced energy supplementation in pregnancy [LiST default]	0	0	0	0
Exclusive breastfeeding: Breastfeeding promotion [33, 41].	30	45.8	88.6	61.1
Complementary feeding- Supplementary feeding and education [33] [author’s calculation]	29.20	45.6	21.5	28.9
Vitamin A (6–59 months) Supplementation [33]	80	66.8	94.6	84.70
Preventive zinc supplementation [LiST default]	0	0	0	0
Management of SAM [42, author’s calculation]	44	14.44	37.29	47.73
Management of MAM [author’s calculation]	20.33	7.82	44.20	20.33

LiST is widely used to model the effect of the scale up of nutrition interventions on stunting and wasting and is explicitly designed for that purpose. LiST lacks default nationally representative data on coverage of many key nutrition interventions and does not account for interactions between wasting and stunting. The first limitation can be bypassed by adding custom values on coverage [35].

We converted the change in stunting rate to the shift in z-score, given that the evidence suggest that it is improved growth, not only the avoidance of stunting, which is associated with better outcomes over the life-course [4, 29, 36].

The estimated increases in z-scores were inputted into an Excel model of the Burkina Faso school system and labour market. The model, using available data on the primary and secondary school system, and data from the literature on the impact of improved nutrition and cash transfers, estimated the present value of life-time earnings under the following scenarios: Scenario 1 (baseline): current level of nutrition intervention, with no new complementary interventions; Scenario 2: expanded access to nutrition interventions to 80% coverage, with no new complementary interventions; and Scenario 3: expanded access to nutrition interventions to 80% coverage, with cash transfers included as a complementary intervention.

The difference between scenarios 1 and 2 provides an estimate of the return on nutrition interventions alone. The difference between 1 and 3 reflects the return on the combined package of nutrition interventions with the cash transfer. The differences are determined from the modelled assumption that a standard deviation improvement in z-score leads to 0.48 additional years of schooling [4, 29, 37], and that the complementary intervention, cash transfers, reduces the school dropout rate by 30% [34]. The scenarios were run for the national and regional birth cohorts. The regional models were calibrated with education data from the regions to reflect the differences in context. The model assumptions and data sources are listed in S1–S5.Texts.

### Cost

LiST was used to estimate the implementation cost of improving nutrition intervention coverage in each model from the perspective of the provider, using existing infrastructure and facilities. The cost estimate is the present value of expanding access to 80% for each of the 10 interventions for a birth cohort. For interventions which are already provided to more than 80%, no change or costs were included. In order to reflect the regional disparities in health care, we added the cost of transport vouchers for the proportion of the population nationally and in each region that report geographic difficulty in accessing clinic and hospital health services (Tables G-K in S4 Text). The costs are based on receiving one voucher per clinic or hospital visit for the number of visits required at 80% coverage of each scenario. Evidence on the effectiveness of transport vouchers is available for Burkina Faso [38]. The cost of the cash transfer is based on the same randomised control trial from which we obtained effectiveness data [34].

The productivity returns nationally and, in each region, were then compared to estimates of the present value of the cost of providing the nutrition interventions and the complementary interventions to the cohort. The total cost and the cost benefit ratio are reported, the latter providing an estimate of the rate of return on investment.

## Results

Table 3 provides an overview of the results. The estimated impact on stunting rates of scaling the 10 evidenced based interventions to 80% coverage is reported (or current coverage if over 80%), along with the implied shift in the mean z-score; data are presented nationally and for each of the regions modelled. These results show a smaller percentage point impact on stunting at the national level and in the Centre compared to the Nord and Sahel. The difference in the implied impact on the z-score is similar.

**Table 3:** Impact, productivity returns and costs of implementing nutrition interventions alone and in combination with cash transfers.

	National	Centre	Nord	Sahel
Additional lives saved children 0–59 mo—expansion of 10 services to 80% in 2023–2027	32 452	2 881	2 536	4 515
Stunting—no new services	25.76	12.90	28.24	44.62
Stunting—expansion of 10 services to 80%	22.98	11.26	25.26	40.77
Change in z-score	0.0887	0.0817	0.0906	0.0982
Scenario 2: Present value of increase in income—nutrition interventions only (per child)	$54 314 793 ($69)	$8 089 562 ($104)	$3 129 295($45)	$1 648 289 ($26)
Scenario 3: Present value of increase in income—nutrition and cash transfer interventions (per child)	$ 1 571 121 053 ($2009)	$167 372 813 ($2146)	$110 973 932 ($1585)	$18 261 061 ($285)
Present Value Cost: nutrition	$54 273 660	$4 775 886	$4 875 110	$4 953 238
Present Value Cost: cash transfers	$81 328 000	$10 140 000	$9 100 000	$8 320 000
Total cost (present value)	$135 601 659	$14 915 886	$13 975 110	$13 273 238
Scenario 2: Cost benefit ratio—nutrition only	1:1	1:2	1:1	1:<0.2
Scenario 3: Cost benefit ratio—combined	1:12	1:11	1:8	1:1

We estimate that the present value of increases in future earnings for a single birth cohort associated with scaling up the nutrition interventions is $54 million, implying an average increase in the present value of lifetime earnings of $69 per child. The total and average per child increases vary by region with the largest improvement expected in the Centre region and the smallest in the Sahel.

The combination of nutrition and cash transfers leads to larger benefits: $1.6 billion at the national level. The per child benefits are still unevenly distributed, although the gap between the Centre and the Nord is less pronounced.

## Discussion

The results from our model suggest that investing in nutrition interventions alone will yield benefits, but the benefits will be distributed according to existing patterns of access to the services required to realize and utilize them, such as access to schooling and job opportunities. In the Centre region, where a higher proportion of children are in school, the long-term benefits of nutrition interventions per child will be the largest. This is true despite the improvement in stunting being the smallest. By comparison, the Sahel region, where stunting is most prevalent, the value of the nutrition interventions to children will be the smallest, as children there lack the opportunities needed to realize or utilize the potential gains through schooling and employment respectively. Including cash transfers in the model alters the outcomes. As expected, the benefits of the combined package are far larger. Moreover, the distribution of benefits no longer so closely reflects the existing pattern of inequality. Indeed, the largest benefit per child would accrue in the Nord.

The cost benefit ratio at the national level suggests that every $1 invested in early nutrition will generate benefits with a present value of $1. This implies that such interventions are worthwhile. Moreover, we should recall that we quantified in financial terms only one aspect of the benefit: productivity returns. Interventions in early nutrition will lead to a host of other benefits, including reduced mortality, estimated at over 32 000 lives saved at the national level, given the association of undernutrition with child mortality [43, 44]. The cost benefit ratios differ by region, with the worst-off region yielding the lowest ratio with a return of less than 0.2 for every dollar spent. If the regional rates of return were used to prioritise investments, they would lead to a heavy bias to the Centre region. This is problematic as it is the region with the lowest levels of stunting. It is noteworthy that the return at the national level is low relative to the returns expected elsewhere in Africa and it is much lower than returns beyond the continent [45–47]. This is because of the poor state of services across the country, including relative to many other countries in Africa. The cost benefit ratios are higher for the combined nutrition plus cash transfer scenario, 1:12 at the national level. There are still regional variations. The results suggest that the cash transfers go a long way to closing the gap between the rate of return in the Centre and Nord regions but do little for the Sahel. This suggests that much more investment in complementary interventions may be required for children in the Sahel to realize the benefits of improved nutrition. The model has several limitations, including limited data availability and a long-time horizon which introduces unavoidable uncertainty. Data was limited to what was publicly available. The publicly available data had gaps making it necessary to use default values in LiST for a number of important assumptions. These limitations should lead to caution when interpreting the estimated value of returns. However, even with the limitations, we can be confident in the distribution of benefits, i.e. that without complementary interventions, a greater proportion of the benefits of nutrition interventions will be realized in better-off regions, reinforcing existing patterns of inequality. It is this pattern which has important implications for priority setting, particularly in the context of a fragile state.

Our analysis is further limited by our modelling the impact of nutrition specific interventions and not nutrition sensitive approaches, and only one complementary intervention. In Burkina Faso there are clearly opportunities to generate large improvement in nutrition outcomes via nutrition sensitive interventions, especially in the regions with limited capacity to deliver nutrition specific services as a consequence of poor infrastructure. While we note these and would encourage their implementation, the caveat that the benefits of these will be constrained (unevenly), by context, still holds. There is also clearly scope for a range of complementary interventions beyond cash transfers. Modelling the impact of these would likely reinforce our argument that the unequal realisation of benefits can be mitigated by later life interventions.

Given that interventions which seek to improve multiple outcomes need to ensure the protection of human development, its realization, and its utilization, we need to identify the rate limiting factors over the life course. Critically, the rate limiting factors may differ. While we highlighted regional differences, we should not forget that rate limiting factors may further differ by sub-population, by gender, or socio-economic status within a region, for example.

The possibility of different rates of return and rate limiting factors by region (or sub-population) underscores the need for a functional government at the national and local level able and willing to intervene. Government along with engagement with civil society and other stakeholders, may often be critical if the right combination of interventions is to be identified.

The bi-directional relationship between human development and state resilience presents a challenge for donors because where the state is needed the most it is typically the weakest. Fragile states, such as Burkina Faso can lack the legitimacy or capacity to effectively set priorities and implement interventions. In such cases, donors sometimes bypass the state entirely and fund NGOs directly [48]. When intervention efforts bypass the state, even if due to state disfunction, opportunities are missed to improve state resilience by strengthen the ability of the state to deliver services and ensuring ownership of the priority setting process which helps to legitimise the state [44, 49]. This challenge has long been recognised and the need for a balanced response by donors highlighted. Newbrander et al, for example, have argued that “[t]here are various options for financing and models of engagement, but support should always combine short-term relief with longer-term development. Stakeholders should aim not only to save lives and protect health but also to bolster nations’ ability to deliver good-quality services in the long run” [50].

## Conclusion

Our model and results suggest that it is not transformative to prioritise investments in a siloed manner in a context where there are multiple constraints on human development. While we examined this with a case study of maternal and child nutrition interventions plus cash-transfer leading to increases in income, we should expect similar patterns with any aspect of human development which builds over the life-course, including health and education.

Taking a life-course perspective draws attention to constraints on human development and thus highlights opportunities to address these constraints. If these opportunities are taken, they will enhance the value of interventions in early nutrition, and in so doing, reduce the inequality in realized benefits. Following such a balanced approach has been shown to improve human development outcomes while building a more resilient state [51, 52]. What our models add to this debate is to point to the risks which arise when, in fragile contexts, donors target a narrow set of outcomes. If they are committed to balancing short and long term demands, a life-course perspective focused on human development is critical. If these opportunities are not taken, interventions to improve early nutrition may act to entrench inequalities. Where these inequalities drive social instability, such entrenchment may act to counter efforts to stabilize the state. Taking a life course perspective and appreciating that the value of interventions is determined by the environments people experience throughout their lives has implications beyond nutrition and fragile contexts. Amartya Sen, argues strongly that you cannot measure wellbeing through income, because what someone can do with a given income differs [53]. This analysis underscores that the same is true of health and development outcomes. The same outcome is of different value to different people. Pushing for the improvement of a single outcome or even a narrowly defined set of outcomes, runs the risk of reinforcing existing inequalities. Geographical, gendered, racial, physical ability and socio-economic differences in life chances shape the value of these outcomes. Focusing on single health, education or other human development outcomes without considering the importance of context over the life-course will, especially in contexts characterised by multiple adversities, lead to risks and missed opportunities.

## Supporting information

S1 Text Projection baseline parameters. (DOCX)

S2 Text Authors’ calculations for regional inputs. (DOCX)

S3 Text Key interventions scaled to 80% each year after baseline. (DOCX)

S4 Text Costing assumptions. (DOCX)

S5 Text Productivity estimates. (DOCX)
