# Supplementary material for: Investing in human development and building state resilience in fragile contexts: A case study of early nutrition investments in Burkina Faso
Source: PLOS Glob Public Health. 2023 Mar 29;3(3):e0001737. doi: 10.1371/journal.pgph.0001737 (PMC10058088; doi:10.1371/journal.pgph.0001737)
Supplement: S1 Text — (DOCX) [file pgph.0001737.s001.docx]

# S1: Projection baseline parametres

All parameters not specified here are default values for Burkina Faso.

### Table A: Modules Overview

| MODULE | DEFAULT DATA SOURCE |
| --- | --- |
| DEMPROJ | WPP 2019 |
| AIM | UNAIDS 2929 |
| FamPlan | Source year: 2014 |
| LIST | DHS 2019 |
| LIST Costing | DHS 2019 |

### Table B: FamPlan Module Inputs

| INPUT | DATA |
| --- | --- |
| Configuration Proximate determinates | checked |

### Table C: Demproj Module Inputs

| INPUT | DATA |
| --- | --- |
| First year | 2022 |
| Final year | 2027 |
| First year population by single year of age 0-80 | Default |
| Sex ratio at birth | Default |
| Total fertility ratio | Default |

### Table D: List Module National Inputs

| INPUT | DATA |
| --- | --- |
| First year of intervention program | 2022 |
| First year of costing | 2022 |
| Display sensitivity bounds | Unchecked |
| Direct entry of stunting | Unchecked |
| Direct entry of wasting | Unchecked |
| Direct entry of fertility risks | Checked |

### Table E: Currency and Inflation

| CURRENCY AND INFLATION | 2022 | 2023 | 2024 | 2025 | 2026 | 2027 |
| --- | --- | --- | --- | --- | --- | --- |
| Exchange rate (XOF-USD) | 582,09 | 582,09 | 582,09 | 582,09 | 582,09 | 582,09 |
| Domestic inflation rate | 0 | 0 | 0 | 0 | 0 | 0 |
| USD inflation rate | 0 | 0 | 0 | 0 | 0 | 0 |

### Table F: National Assumptions

| PARAMETER | | LIST DEFAULT ASSUMPTION | | | BASELINE ASSUMPTION | | |
| --- | --- | --- | --- | --- | --- | --- | --- |
|  |  | **DEFINITION** | **FIG** | **SOURCE** | **DEFINITION** | **FIG** | **SOURCE** |
| HEALTH, HOUSEHOLD AND ECONOMIC STATUS | | | | | | | |
| Average household size, no. of persons per household | The average no. of people living in a household. | | **6** | Data are drawn from DHS, MICS, and other nationally representative household surveys. | Mean no. of household members | **5,70** | Institut National de la Statistique et de la Démographie (INSD), Programme d’Appui au Développement Sanitaire (PADS), Programme National de Lutte contre le Paludisme (PNLP et ICF). Enquête sur les indicateurs du paludisme au Burkina Faso, 2017-2018. Rockville, Maryland: 2018. https://dhsprogram.com/pubs/pdf/MIS32/MIS32.pdf |
| Baseline child mortality, Neonatal mortality rate | No. of deaths during the neonatal period (first 28 completed days of life) per 1,000 live births. | | **25,94** | IGME estimates for years 1996-2019 (www.childmortality.org) | Neonatal mortality rate is the no. of neonates dying before reaching 28 days of age, per 1,000 live births in a given year, 2020 | **25,80** | Estimates developed by the UN Inter-agency Group for Child Mortality Estimation (UNICEF, WHO, World Bank, UN DESA Population Division) at [www.childmortality.org](http://www.childmortality.org). |
| Baseline child mortality, infant mortality | No. of deaths of children under one year of age per 1,000 live births | | **53,94** | IGME estimates for years 1996-2019 (www.childmortality.org) | No. of infants dying before reaching one year of age, per 1,000 live births in a given year, 2020 | **52,8** | Estimates developed by the UN Inter-agency Group for Child Mortality Estimation (UNICEF, WHO, World Bank, UN DESA Population Division) at [www.childmortality.org](http://www.childmortality.org). |
| Baseline child mortality, child mortality rate | Probability of a child born in a specific year or period dying before reaching the age of five, if subject to age-specific mortality rates of that period. | | **87,54** | IGME estimates for years 1996-2019 (www.childmortality.org) | Probability per 1,000 that a new-born baby will die before reaching age five, if subject to age-specific mortality rates of the specified year, 2020 | **85,00** | Estimates developed by the UN Inter-agency Group for Child Mortality Estimation (UNICEF, WHO, World Bank, UN DESA Population Division) at [www.childmortality.org](http://www.childmortality.org). |
| Baseline maternal mortality | Maternal mortality ratio (maternal deaths per 100,000 live births) | | **320** | WHO, UNICEF, UNFPA, World Bank Group and the United Nations Population Division. Trends in maternal mortality: 1990 to 2015. |  | **330** | Ministère de la Santé (MOH). *Enquête Nutritionnelle Nationale* 2019. Burkina Faso: MOH. |
| Baseline maternal health, Prevalence of low BMI among women of reproductive age | % women aged 15-49 with BMI <18.5 | | **14,74** | Fincane, MM. et al. National, regional, and global trends in body-mass index since 1980: Systematic analysis of health examination surveys and epidemiological studies with 960 country-years and 9.1 million participants. *Lancet*. 2011;377:557–67. | Nutritional status in women of childbearing age according to BMI <18.5 % | **9** | Ministère de la Santé (MOH). 2020. *Enquête Nutritionnelle Nationale* *Rapport Final (SMART 2020).* Burkina Faso: MOH. |
| Percent of pregnant women with anaemia | Percent of pregnant women (Hb <110 g/L) | | **57.5** | Stevens GA, et al. Global, regional, and national trends in haemoglobin concentration and prevalence of total and severe anaemia in children and pregnant for 1995-2011: a systematic analysis of population-representative data. *Lancet Global Health* 2013; 1(1): e16-25. | Anaemia in pregnant women (Hb <110 g/L) (%) 2016 | **57,5** | World health Organisation. Global Health Observatory (GHO). EHOhttp://apps.who.int/ghodata/ |
| Nutrition status distributions single indicator of stunting | Stunting (more than 2 SD less than the median norm) | | **35** | DHS 1998, DHS 2003, MICS 2006, DHS 2010 | 2019, % children 0-59 months of age (Height/Age z–Score <–2) | **24,9** | Ministère de la Santé (MOH). 2020. *Enquête Nutritionnelle Nationale* *Rapport Final (SMART 2020).* Burkina Faso: MOH. |
| Nutrition status distributions single indicator of wasting | Wasting (more than 2 SD less than the median norm) | | **15,5** | DHS 1998, DHS 2003, MICS 2006, DHS 2010 | 2019, % children 6-59 months of age (Weight/Height z–score <–2) | **9,1** | Ministère de la Santé (MOH). 2020. *Enquête Nutritionnelle Nationale* *Rapport Final (SMART 2020).* Burkina Faso: MOH. |
| COVERAGE | | | | | | | |
| Complementary feeding -education only | % mothers intensively counseled on the importance of continued breastfeeding beyond six months and appropriate complementary feeding practices. As a proxy, the % 6-23 month old children receiving minimum dietary diversity (4+ food groups) is used. | | **5,2** | DHS 2010 | 2017, % mothers of children 6-11 months of age, who received advice on complementary feeding in the last 30 days | **26** | Institut National de la Statistique et de la Démographie (INSD), and The Bill & Melinda Gates Institute for Population and Reproductive Health at The Johns Hopkins Bloomberg School of Public Health. 2018. *Performance Monitoring and Accountability 2020 (PMA2020) Survey Round 6, PMA2018/Burkina Faso-R6*. Ouagadougou, Burkina Faso and Baltimore, Maryland, USA​. |
| Prevention of malaria in pregnancy | % Pregnant women receiving 2+ doses of Sp/Fansidar or sleeping under an insecticide treated net during pregnancy. | | **47,6** | MICS 2006, DHS 2010, MIS 2014 | Proportion of pregnant women who received IPT3 during ANC | **55,9** | https://burkinafaso.opendataforafrica.org/xbsbqhb/sous-nutrition?regionId=BF-12  accessed 29-03-22 |
| Utilisation ANC at least 4 visits | ANC at least 4 visits | | **33,1** | Coverage data for this indicator are drawn from DHS, MICS, and other nationally representative household surveys. | % Women who recently gave birth who went to 4 ANC contacts, 2017 | **67** | Alive & Thrive. 2021. *Nutrition Interventions in Antenatal Care and Immediate Postnatal Care in Burkina Faso: Findings from a baseline survey in Burkina Faso*. |
| Prevalence of early initiation of breastfeeding | % children who begin breastfeeding within 1 hour of birth. | | **42,1** | DHS 1998, DHS 2003, MICS 2006, DHS 2010 | % Women who practiced early initiation breastfeeding within the hour birth tracking | **63,4** | Ministère de la Santé (MOH). 2020. *Enquête Nutritionnelle Nationale Rapport Final (SMART 2020).* Burkina Faso: MOH. |
| Prevalence of any breastfeeding 12-23 months | % children still receiving any breastmilk. | | **90,5** | DHS 1998, DHS 2003, MICS 2006, DHS 2010 | % Children 12-23 months of age who were fed with breastmilk during the previous day. | **92** | United Nations Children’s Fund (UNICEF). 2021. The State of the World’s Children 2021. New York: UNICEF. |
| KEY INTERVENTION COVERAGE | | | | | | | |
| Folic acid supplementation in pregnancy | % women 15-49 that have appropriate food fortification (0.4 mg folic acid per day) around the time of pregnancy. | | **14,5** | Food Fortification Initiative http://www.ffinetwork.org/country-profiles | - | **14,5** | Default |
| Multiple micronutrient supplementation in pregnancy  *Micronutrient supplementation in pregnancy* | % pregnant women taking a multiple micronutrient supplement daily. A multiple micronutrient supplement is defined as a supplement  containing at least iron, folate, and additional vitamins/minerals. | | **0** | Coverage data for this indicator are not typically available. Currently set at 0 for baseline; user should enter local data if possible and available | Multiple micronutrients in routine ANC services | **0** | Sanghvi, T. et al (2021). Gaps in the implementation and uptake of maternal nutrition interventions in antenatal care services in Bangladesh, Burkina Faso, Ethiopia and India. *Maternal & Child Nutrition*, p.e13293. |
| *Iron supplementation in pregnancy* | % pregnant women taking an iron supplement daily, for at least 90 days. | | **50,2** | DHS 2003, DHS 2010 | Women who were recently pregnant who took at least 90-180 tablets  iron/folic acid, 2017 | **67.4** | Ministère de la Santé (MOH). 2020. *Enquête Nutritionnelle Nationale* *Rapport Final (SMART 2020).* Burkina Faso: MOH. |
| Calcium supplementation in pregnancy | % Pregnant women taking 1g of calcium daily | | **0** | Coverage data for this indicator are not typically available. Currently set at 0 for baseline; user should enter local data if possible and available | Maternal nutrition guidelines adopted in 2017 by the Ministry of Health excluded calcium supplementation | **0** | Alive & Thrive 2018. Renforcement de la nutrition de la mère et l’alimentation du nourrisson et du jeune enfant dans les plateformes de santé de la reproduction, de la mère, du nouveau-né, de l’enfant au Burkina Faso : un besoin urgent. https://www.aliveandthrive.org/sites/default/files/attachments/RMNCH-brief-French.pdf |
| Balanced energy supplementation in pregnancy | % pregnant women who are food insecure who receive balanced energy-protein (BEP) supplementation. | | **0** | Coverage data for this indicator are not typically available. Currently set at 0 for baseline; user should enter local data if possible and available | - | **0** | Default |
| Exclusive breastfeeding: Breastfeeding promotion | % children whose mothers receive activities designed to promote breastfeeding. Breastfeeding promotion can either be one-on-one or group meetings. Promotion activities can take place within the health system. Health system interventions include the Baby-Friendly Hospital Initiative, establishment of rooming-in practices, organizational support on breastfeeding outcomes, etc. | | **22,1** | Coverage data for this indicator are not typically available. As a proxy, the level of breastfeeding promotion is set by default to equal % children 1-5 months of age that are exclusively breastfed; user should enter local data if possible and available. | % Women who received breastfeeding counselling | **30** | Alive & Thrive. 2021. *Nutrition Interventions in Antenatal Care and Immediate Postnatal Care in Burkina Faso: Findings from a baseline survey in Burkina Faso*. |
| Complementary feeding- Supplementary feeding and education | % mothers intensively counselled on the importance of continued breastfeeding beyond six months and appropriate complementary feeding practices, and given appropriate dietary supplementation. As a proxy, the % 6-23 month old children receiving minimum dietary diversity (4+ food groups) is used. | | **5,2** | DHS 2010 | % Children 6-23 months of age receiving minimum dietary diversity (4+ food groups) | **29,20** | Ministère de la Santé (MOH). 2020. *Enquête Nutritionnelle Nationale* *Rapport Final (SMART 2020).* Burkina Faso: MOH. |
| Vitamin A (6- 59 months) Supplementation | % children 6-59 months of age receiving two doses of Vitamin A during the last 12 months. | | **99** | UNICEF - Vitamin A coverage. http://data.unicef.org/nutrition/vitamin-a. Updated annually. | % Children 6-59 months of age who were supplemented with vitamin A during the last six months, 2019 | **80** | Ministère de la Santé (MOH). 2020. *Enquête Nutritionnelle Nationale* Rapport Final: SMART -2020. |
| Preventive zinc supplementation | % children 12-59 months of age who are given daily supplements of 10mg zinc. | | **0** | Coverage data for this indicator are not typically available. Currently set at 0 for baseline. | - | **0** | Default |
| Management of SAM | % severely wasted children (<-3 Z-score) receiving therapeutic feeding. Therapeutic feeding is outpatient treatment including supplementation with ready-to-use therapeutic foods (RUTF) and maternal education. | | **26,1** | Coverage estimates calculated from: The UNICEF Global SAM Management Update Tool (NutriDash): 2012 – 2016. Available at www.acutemalnutrition.org.The UNICEF-WHO-The World Bank Group Joint Child Malnutrition Estimates: 2012 – 2016. Available at www.acutemalnutrition.org. | Treatment  coverage (related to estimated burden), 2018 | **44** | United Nations Children’s Fund (UNICEF). 2018. *Sahel – SAM monthly factsheet 2018 Report covering January to December* 2018. UNICEF. |
| Management of MAM | % moderately wasted children (-3 to -2 Z-score) receiving outpatient treatment including supplementation with RUSF and maternal education. | | **0** | Coverage data for this indicator are not typically available. Currently set at 0 for baseline. | Authors’ calculation | **20,33** | Authors’ calculation |

### Table G: Subnational Wizard Assumptions

| PARAMETER | NORD | CENTRE | SAHEL | DATA SOURCE | DEFINITION |
| --- | --- | --- | --- | --- | --- |
| DEMPROJ MODULE | | | | | |
| National population | 21874181 | 21874181 | 21874181 | Default | 2022 |
| Subnational population | 1 837431 | 3 237 379 | 1397760 | Default calculation based on percentage | 2022 |
| % Of national population | 8,40 | 14,80 | 5,34 | Fifth General Population and Housing Census of Burkina Faso, 2019. <https://burkinafaso.opendataforafrica.org/> |  |
| Total Fertility rate | 5,28 | 3,25 | 6,39 | Authors’ calculation |  |
| AIM MODULE | | | | | |
| HIV Incidence | - | - | - | Default | 2022 |
| PMTCT | - | - | - | Default | 2022 |
| Cotrimoxazole | - | - | - | Default | 2022 |
| Adult ART | - | - | - | Default | 2022 |
| LIST MODULE | | | | | |
| NATIONAL (No subnational data available, national is default) | | | | |  |
| % Vitamin A deficient | - | - | - | Default | 2022 |
| % Zinc deficient | - | - | - | Default | 2022 |
| SUBNATIONAL | | | | |  |
| Stunting distributions (single indicator) | 27,3 | 12,5 | 43,1 | Ministère de la Santé (MOH). 2020. *Enquête Nutritionnelle Nationale* Rapport Final: SMART -2020. | % Children 0-59 months of age (Height/Age z–Score <–2), 2019 |
| Wasting distributions (single indicator) | 8,2 | 8,5 | 15,1 | Ministère de la Santé (MOH). 2020. *Enquête Nutritionnelle Nationale* Rapport Final: SMART -2020. | % Children 6-59 months of age (Weight/Height z–score <–2), 2019 |
| Intervention coverage (all other interventions not specified below are default) | | | | | |
| Folic acid fortification | 14,5 | 14,5 | 14,5 | Default | 2022 |
| Micronutrient supplementation in pregnancy | 0 | 0 | 0 | Sanghvi, T. et al (2021). Gaps in the implementation and uptake of maternal nutrition interventions in antenatal care services in Bangladesh, Burkina Faso, Ethiopia and India. *Maternal & Child Nutrition*, p.e13293. |  |
| Iron supplementation in pregnancy | 73.1 | 65.9 | 45.6 | Ministère de la Santé (MOH). 2020. *Enquête Nutritionnelle Nationale* *Rapport Final (SMART 2020).* Burkina Faso: MOH. | Proportion women of reproductive age who took between 90-180 iron/folic acid tablets (95% CI) during their last pregnancy |
| Calcium supplementation in pregnancy | 0 | 0 | 0 | Default | 2022 |
| Balanced energy supplementation in pregnancy | 0 | 0 | 0 | Default | 2022 |
| Breastfeeding promotion *(manually adjusted post wizard)* | 88,6 | 45,8 | 61,1 | Ministère de la Santé (MOH). 2020. *Enquête Nutritionnelle Nationale* *Rapport Final (SMART 2020).* Burkina Faso: MOH. | Under 6-month exclusive breast feeding |
| Prevalence of Early initiation of Breastfeeding | 71,80 | 49,6 | 27 | Ministère de la Santé (MOH). 2020. *Enquête Nutritionnelle Nationale* *Rapport Final (SMART 2020).* Burkina Faso: MOH. |  |
| Continued breastfeeding (any breastfeeding 12-23 mo) | 126,77 (rounded down to 100) | 63,11 | 84,19 | Authors’ calculation |  |
| Complementary feeding -education only | 19,14 | 40,60 | 25,73 | Authors’ calculation |  |
| Complementary feeding Supplementary feeding and education | 21,5 | 45,6 | 28,9 | Ministère de la Santé (MOH). 2020. *Enquête Nutritionnelle Nationale* *Rapport Final (SMART 2020).* Burkina Faso: MOH. | % Children 6–23 months of age receiving minimum dietary diversity (4+ food groups) |
| Vitamin A (6- 59 months) Supplementation | 94,6 | 66,8 | 84,70 | Ministère de la Santé (MOH). 2020. *Enquête Nutritionnelle Nationale* *Rapport Final (SMART 2020).* Burkina Faso: MOH. |  |
| Preventive zinc supplementation | 0 | 0 | 0 | Default | 2022 |
| Management of SAM | 37,29 | 14,44 | 47,73 | Authors’ calculation |  |
| Management of MAM | 44,20 | 7,82 | 20,33 | Authors’ calculation |  |
| CALCULATED (Default, Indicators calculated on coverage differentials) | | | | | |
| Neonatal, infant and under 5 mortality rate | - | - | - | Default | 2022 |
| Neonatal and post neonatal causes of death | - | - | - | Default | 2022 |
| Stillbirth rate | - | - | - | Default | 2022 |
| Still birth causes | - | - | - | Default | 2022 |
| Maternal mortality ratio | - | - | - | Default | 2022 |
| Maternal causes of death | - | - | - | Default | 2022 |
| FAMPLAN MODULE | | | | | |
| Contraceptive prevalence rate | - | - | - | Default | 2022 |

“For subnational projections at the state level, we used the Subnational Wizard from the LiST to input available state-level data on population, total fertility rate, stunting and wasting distributions, breastfeeding practices and several intervention coverages. For inputs that were not available at subnational areas, the Wizard uses the LiST to project the missing data based on the difference between national and subnational intervention coverage.” The national inputs in subnational wizard stays default.
