# Supplementary material for: Investing in human development and building state resilience in fragile contexts: A case study of early nutrition investments in Burkina Faso
Source: PLOS Glob Public Health. 2023 Mar 29;3(3):e0001737. doi: 10.1371/journal.pgph.0001737 (PMC10058088; doi:10.1371/journal.pgph.0001737)
Supplement: S2 Text — (DOCX) [file pgph.0001737.s002.docx]

# S2: Authors’ Calculations for Regional Inputs

### Table A: Complementary Feeding-Education Only

| DEFINITION | SOURCE | NATIONAL | SAHEL | CENTRE | NORTH |
| --- | --- | --- | --- | --- | --- |
| Complementary feeding - supplementary feeding and education | Ministère de la Santé (MOH). 2020. *Enquête Nutritionnelle Nationale* *Rapport Final (SMART 2020).* Burkina Faso: MOH. | 29,20 | 28,9 | 45,6 | 21,5 |
| Complementary feeding -education only | Institut National de la Statistique et de la Démographie (INSD), and The Bill & Melinda Gates Institute for Population and Reproductive Health at The Johns Hopkins Bloomberg School of Public Health. 2018. *Performance Monitoring and Accountability 2020 (PMA2020) Survey Round 6, PMA2018/Burkina Faso-R6*. Ouagadougou, Burkina Faso and Baltimore, Maryland, USA​. | 26,00 | - | - | - |
| National complementary feeding - education only as proportion of Complementary feeding - supplementary feeding and education | - | 0,89 | - | - | - |
| Complementary feeding -education only | - |  | 25,73 | 40,60 | 19,14 |

### Table B: SAM Treatment

| DEFINITION | SOURCE | NATIONAL | SAHEL | CENTRE | NORTH |
| --- | --- | --- | --- | --- | --- |
| Screening rate for severe acute malnutrition (SAM) 2020 | Institut National de la Statistique et de la Démographie (INSD). 2020. *Enquête nationale et routine 2009-2020.* Burkina Faso: INSD. | 64,90 | 70,40 | 21,30 | 55,00 |
| SAM treatment | United Nations Children’s Fund (UNICEF). 2018. *Sahel – SAM monthly factsheet 2018 Report covering January to December* 2018. UNICEF. | 44,00 | - | - |  |
| National SAM treatment as proportion of National SAM screening rate | - | 0,68 | - | - | - |
| SAM treatment | - |  | 47,73 | 14,44 | 37,29 |

### Table C: MAM Treatment

| DEFINITION |  | NATIONAL | SAHEL | CENTRE | NORTH |
| --- | --- | --- | --- | --- | --- |
| Screening rate for moderate acute malnutrition (MAM) 2020 | Institut National de la Statistique et de la Démographie (INSD). 2020. *Enquête nationale et routine 2009-2020.* Burkina Faso: INSD. | 29,90 | 85,00 | 11,50 | 65,00 |
| SAM treatment as proportion of SAM screening rate | - | 0,68 | - | - | - |
| MAM treatment (national SAM treatment as proportion of national SAM screening x Regional MAM screening rate) | - | 20,33 | 57,80 | 7,82 | 44,20 |

### Table D: Total Fertility Rate

| DEFINITION | SOURCE | NATIONAL | SAHEL | CENTRE | NORTH |
| --- | --- | --- | --- | --- | --- |
| Total fertility rate represents the number of children that would be born to a woman if she were to live to the end of her childbearing years and bear children in accordance with age-specific fertility rates of the specified year, 2019 | The World Bank. Fertility rate, total (births per woman). The World Bank Group. | 5,11 | - | - | - |
| TFR 2010 |  | 6,00 | 7,50 | 3,70 | 6,20 |
| Regional TFR 2010 as proportion of national TFR | - |  | 1,25 | 0,62 | 1,03 |
| TRR 2019 per region | - |  | 6,39 | 3,15 | 5,28 |
