# Supplementary material for: Investing in human development and building state resilience in fragile contexts: A case study of early nutrition investments in Burkina Faso
Source: PLOS Glob Public Health. 2023 Mar 29;3(3):e0001737. doi: 10.1371/journal.pgph.0001737 (PMC10058088; doi:10.1371/journal.pgph.0001737)
Supplement: S3 Text — (DOCX) [file pgph.0001737.s003.docx]

# S3: Scenario Key Interventions Scaled to 80% Each Year After Baseline

## SUMMARY STUNTING

### Table A: Global Stunting (<-2 SD) Rate 2022-2027

| 24-59 MONTHS | BASELINE 2027 | SCENARIO 2027 |
| --- | --- | --- |
| NATIONAL | 29,03 | 24,74 |
| NORD | 31,85 | 27,25 |
| CENTRE | 14,43 | 11,95 |
| SAHEL | 50,43 | 44,75 |

## NATIONAL 80% COVERAGE SCENARIO

### Table B: National Coverage Summary Key Interventions 2022-2027

| INTERVENTIONS | BASELINE | | | | | | 80% SCALE UP SCENARIO | | | | | |
| --- | --- | --- | --- | --- | --- | --- | --- | --- | --- | --- | --- | --- |
|  | **2022** | **2023** | **2024** | **2025** | **2026** | **2027** | **2022** | **2023** | **2024** | **2025** | **2026** | **2027** |
| Folic acid fortification/ supp | 14,5 | 14,5 | 14,5 | 14,5 | 14,5 | 14,5 | 14,5 | 80 | 80 | 80 | 80 | 80 |
| Micronutrient supplementation (iron and multiple micronutrients) | 67,4 | 67,4 | 67,4 | 67,4 | 67,4 | 67,4 | 67,4 | 80 | 80 | 80 | 80 | 80 |
| *Iron supplementation in pregnancy* | *67,4* | *67,4* | *67,4* | *67,4* | *67,4* | *67,4* | *67,4* | *67,4* | *67,4* | *67,4* | *67,4* | *67,4* |
| *Multiple micronutrient supplementation in pregnancy* | *0* | *0* | *0* | *0* | *0* | *0* | *0* | *12,6* | *12,6* | *12,6* | *12,6* | *12,6* |
| Calcium supplementation | 0 | 0 | 0 | 0 | 0 | 0 | 0 | 80 | 80 | 80 | 80 | 80 |
| Balanced energy supplementation | 0 | 0 | 0 | 0 | 0 | 0 | 0 | 80 | 80 | 80 | 80 | 80 |
| Complementary feeding - supplementary feeding and education | 29,2 | 29,2 | 29,2 | 29,2 | 29,2 | 29,2 | 29,2 | 80 | 80 | 80 | 80 | 80 |
| Vitamin A supplementation | 80 | 80 | 80 | 80 | 80 | 80 | 80 | 80 | 80 | 80 | 80 | 80 |
| Zinc supplementation | 0 | 0 | 0 | 0 | 0 | 0 | 0 | 80 | 80 | 80 | 80 | 80 |
| Promotion of breastfeeding | 30 | 30 | 30 | 30 | 30 | 30 | 30 | 80 | 80 | 80 | 80 | 80 |
| SAM - treatment for severe acute malnutrition | 44 | 44 | 44 | 44 | 44 | 44 | 44 | 80 | 80 | 80 | 80 | 80 |
| MAM - treatment for moderate acute malnutrition | 20,3 | 20,3 | 20,3 | 20,3 | 20,3 | 20,3 | 20,3 | 80 | 80 | 80 | 80 | 80 |

### Table C: National Global Stunting (<-2 Sd) Rate 2022-2027

| AGE GROUP | BASELINE | | | | | | 80% COVERAGE SCENARIO | | | | | |
| --- | --- | --- | --- | --- | --- | --- | --- | --- | --- | --- | --- | --- |
|  | 2022 | 2023 | 2024 | 2025 | 2026 | 2027 | 2022 | 2023 | 2024 | 2025 | 2026 | 2027 |
| <1 month | 9,31 | 9,31 | 9,31 | 9,31 | 9,31 | 9,31 | 9,31 | 9,08 | 9,08 | 9,08 | 9,08 | 9,08 |
| 1-5 months | 9,31 | 9,31 | 9,31 | 9,31 | 9,31 | 9,31 | 9,31 | 9,14 | 9,14 | 9,14 | 9,14 | 9,14 |
| 6-11 months | 14,00 | 14,00 | 14,00 | 14,00 | 14,00 | 14,00 | 14,00 | 13,50 | 13,50 | 13,50 | 13,50 | 13,50 |
| 12-23 months | 25,76 | 25,76 | 25,76 | 25,76 | 25,76 | 25,76 | 25,76 | 23,35 | 22,98 | 22,98 | 22,98 | 22,98 |
| 24-59 months | 29,03 | 29,03 | 29,03 | 29,03 | 29,03 | 29,03 | 29,03 | 26,96 | 26,32 | 25,58 | 24,84 | 24,74 |
| Total (0-59 months) | 24,58 | 24,57 | 24,57 | 24,58 | 24,57 | 24,57 | 24,58 | 22,82 | 22,38 | 21,96 | 21,53 | 21,47 |

### Table D: National No. Of Stunting Cases Averted by Intervention (Total (0-59mo)) 2022-2027

| INTERVENTION | 80% COVERAGE SCENARIO | | | | | |
| --- | --- | --- | --- | --- | --- | --- |
|  | 2022 | 2023 | 2024 | 2025 | 2026 | 2027 |
| Calcium supplementation | 0 | 1 363 | 2 648 | 3 264 | 3 891 | 4 019 |
| Micronutrient supplementation (iron & MMN) | 0 | 498 | 967 | 1 193 | 1 422 | 1 469 |
| Balanced energy supplementation | 0 | 532 | 1 034 | 1 274 | 1 519 | 1 569 |
| Age-appropriate breastfeeding practices | 0 | 1 268 | 2 559 | 3 178 | 3 807 | 3 935 |
| Appropriate complementary feeding | 0 | 5 389 | 11 743 | 16 543 | 21 412 | 22 306 |
| Zinc supplementation | 0 | 99 174 | 116 070 | 135 962 | 156 294 | 161 167 |

### Table E: National No. Of Additional Lives Saved (Total (0-59mo)) 2022-2027

| INTERVENTION | 80% COVERAGE SCENARIO | | | | | |
| --- | --- | --- | --- | --- | --- | --- |
|  | 2022 | 2023 | 2024 | 2025 | 2026 | 2027 |
| Folic acid fortification | 0 | 112 | 114 | 115 | 117 | 118 |
| Calcium supplementation | 0 | 291 | 298 | 302 | 306 | 310 |
| Micronutrient supplementation (iron and multiple micronutrients) | 0 | 102 | 104 | 105 | 107 | 108 |
| Balanced energy supplementation | 0 | 199 | 203 | 206 | 209 | 211 |
| Age appropriate breastfeeding practices due to promotion | 0 | 303 | 308 | 312 | 316 | 297 |
| Zinc supplementation | 0 | 1589 | 1635 | 1688 | 1742 | 1770 |
| Complementary feeding via reduction in stunting | 0 | 63 | 99 | 115 | 132 | 136 |
| Complementary feeding via reduction in wasting | 0 | 96 | 98 | 99 | 101 | 102 |
| SAM - treatment for severe acute malnutrition | 0 | 276 | 279 | 283 | 286 | 290 |
| MAM - treatment for moderate acute malnutrition | 0 | 3197 | 3240 | 3280 | 3319 | 3364 |

## NORD 80% COVERAGE SCENARIO

### Table F: Nord Coverage Summary Key Intervention 2022-2027

| INTERVENTIONS | BASELINE | | | | | | 80% SCALE UP SCENARIO | | | | | |
| --- | --- | --- | --- | --- | --- | --- | --- | --- | --- | --- | --- | --- |
|  | 2022 | 2023 | 2024 | 2025 | 2026 | 2027 | 2022 | 2023 | 2024 | 2025 | 2026 | 2027 |
| Folic acid fortification | 14,5 | 14,5 | 14,5 | 14,5 | 14,5 | 14,5 | 14,5 | 80 | 80 | 80 | 80 | 80 |
| Micronutrient supplementation (iron and multiple micronutrients) | 73,1 | 73,1 | 73,1 | 73,1 | 73,1 | 73,1 | 73,1 | 80 | 80 | 80 | 80 | 80 |
| *Iron supplementation in pregnancy* | *73,1* | *73,1* | *73,1* | *73,1* | *73,1* | *73,1* | *73,1* | *73,1* | *73,1* | *73,1* | *73,1* | *73,1* |
| *Multiple micronutrient supplementation in pregnancy* | *0* | *0* | *0* | *0* | *0* | *0* | *0* | *6,9* | *6,9* | *6,9* | *6,9* | *6,9* |
| Calcium supplementation | 0 | 0 | 0 | 0 | 0 | 0 | 0 | 80 | 80 | 80 | 80 | 80 |
| Complementary feeding - supplementary feeding and education | 21,5 | 21,5 | 21,5 | 21,5 | 21,5 | 21,5 | 21,5 | 80 | 80 | 80 | 80 | 80 |
| Vitamin A supplementation | 94,6 | 94,6 | 94,6 | 94,6 | 94,6 | 94,6 | 94,6 | 94,6 | 94,6 | 94,6 | 94,6 | 94,6 |
| Zinc supplementation | 0 | 0 | 0 | 0 | 0 | 0 | 0 | 0 | 80 | 80 | 80 | 80 |
| Promotion of breastfeeding | 88,6 | 88,6 | 88,6 | 88,6 | 88,6 | 88,6 | 88,6 | 88,6 | 88,6 | 88,6 | 88,6 | 88,6 |
| Balanced energy supplementation | 0 | 0 | 0 | 0 | 0 | 0 | 0 | 80 | 80 | 80 | 80 | 80 |
| SAM - treatment for severe acute malnutrition | 37,3 | 37,3 | 37,3 | 37,3 | 37,3 | 37,3 | 37,3 | 80 | 80 | 80 | 80 | 80 |
| MAM - treatment for moderate acute malnutrition | 44,2 | 44,2 | 44,2 | 44,2 | 44,2 | 44,2 | 44,2 | 80 | 80 | 80 | 80 | 80 |

### Table G: Nord Global Stunting (<-2 SD) Rate

| AGE GROUP | BASELINE | | | | | | 80% COVERAGE SCENARIO | | | | | |
| --- | --- | --- | --- | --- | --- | --- | --- | --- | --- | --- | --- | --- |
|  | 2022 | 2023 | 2024 | 2025 | 2026 | 2027 | 2022 | 2023 | 2024 | 2025 | 2026 | 2027 |
| <1 month | 10,12 | 10,12 | 10,12 | 10,12 | 10,12 | 10,12 | 10,12 | 9,89 | 9,89 | 9,89 | 9,89 | 9,89 |
| 1-5 months | 10,12 | 10,12 | 10,12 | 10,12 | 10,12 | 10,12 | 10,12 | 9,98 | 9,98 | 9,98 | 9,98 | 9,98 |
| 6-11 months | 15,29 | 15,29 | 15,29 | 15,29 | 15,29 | 15,29 | 15,29 | 14,74 | 14,74 | 14,74 | 14,74 | 14,74 |
| 12-23 months | 28,24 | 28,24 | 28,24 | 28,24 | 28,24 | 28,24 | 28,24 | 25,66 | 25,26 | 25,26 | 25,26 | 25,26 |
| 24-59 months | 31,85 | 31,85 | 31,85 | 31,85 | 31,85 | 31,85 | 31,85 | 29,64 | 28,95 | 28,15 | 27,35 | 27,25 |
| Total (0-59 months) | 26,86 | 26,90 | 26,97 | 26,98 | 26,96 | 26,89 | 26,86 | 25,01 | 24,61 | 24,15 | 23,68 | 23,57 |

### Table H: Nord No. Of Stunting Cases Averted by Intervention (Total (0-59mo)) 2022-2027

| INTERVENTION | 80% COVERAGE SCENARIO | | | | | |
| --- | --- | --- | --- | --- | --- | --- |
|  | 2022 | 2023 | 2024 | 2025 | 2026 | 2027 |
| Calcium supplementation | 0 | 52 | 111 | 141 | 171 | 177 |
| Micronutrient supplementation (iron & MMN) | 0 | 8 | 18 | 23 | 28 | 29 |
| Balanced energy supplementation | 0 | 42 | 89 | 113 | 138 | 143 |
| Appropriate complementary feeding | 0 | 600 | 1 259 | 1 815 | 2 379 | 2 464 |
| Zinc supplementation | 0 | 5 562 | 6 573 | 7 763 | 8 950 | 9 101 |

### Table I: Nord No. Of Additional Lives Saved by Intervention (Total (0-59mo)) 2022-2027

|  | 80% COVERAGE SCENARIO | | | | | |
| --- | --- | --- | --- | --- | --- | --- |
|  | 2022 | 2023 | 2024 | 2025 | 2026 | 2027 |
| Folic acid fortification | 0 | 9 | 9 | 9 | 10 | 10 |
| Calcium supplementation | 0 | 24 | 24 | 25 | 26 | 26 |
| Micronutrient supplementation (iron and multiple micronutrients) | 0 | 5 | 5 | 5 | 5 | 5 |
| Balanced energy supplementation | 0 | 26 | 27 | 27 | 28 | 29 |
| Zinc supplementation | 0 | 127 | 133 | 138 | 143 | 144 |
| Complementary feeding via reduction in stunting | 0 | 6 | 10 | 12 | 14 | 14 |
| Complementary feeding via reduction in wasting | 0 | 9 | 9 | 9 | 9 | 9 |
| SAM - treatment for severe acute malnutrition | 36 | 36 | 37 | 37 | 38 | 36 |
| MAM - treatment for moderate acute malnutrition | 196 | 196 | 199 | 203 | 206 | 196 |

## CENTRE 80% COVERAGE SCENARIO

### Table J: Centre Coverage Summary Key Intervention 2022-2027

| INTERVENTIONS | BASELINE | | | | | | 80% SCALE UP SCENARIO | | | | | |
| --- | --- | --- | --- | --- | --- | --- | --- | --- | --- | --- | --- | --- |
|  | 2022 | 2023 | 2024 | 2025 | 2026 | 2027 | 2022 | 2023 | 2024 | 2025 | 2026 | 2027 |
| Folic acid fortification | 14,5 | 14,5 | 14,5 | 14,5 | 14,5 | 14,5 | 14,5 | 80 | 80 | 80 | 80 | 80 |
| Micronutrient supplementation (iron and multiple micronutrients) | 65,9 | 65,9 | 65,9 | 65,9 | 65,9 | 65,9 | 65,9 | 80 | 80 | 80 | 80 | 80 |
| *Iron supplementation in pregnancy* | *65,9* | *65,9* | *65,9* | *65,9* | *65,9* | *65,9* | *65,9* | *65,9* | *65,9* | *65,9* | *65,9* | *65,9* |
| *Multiple micronutrient supplementation in pregnancy* | *0* | *0* | *0* | *0* | *0* | *0* | *0* | *14,1* | *14,1* | *14,1* | *14,1* | *14,1* |
| Calcium supplementation | 0 | 0 | 0 | 0 | 0 | 0 | 0 | 80 | 80 | 80 | 80 | 80 |
| Complementary feeding - supplementary feeding and education | 45,6 | 45,6 | 45,6 | 45,6 | 45,6 | 45,6 | 45,6 | 80 | 80 | 80 | 80 | 80 |
| Vitamin A supplementation | 66,8 | 66,8 | 66,8 | 66,8 | 66,8 | 66,8 | 66,8 | 80 | 80 | 80 | 80 | 80 |
| Zinc supplementation | 0 | 0 | 0 | 0 | 0 | 0 | 0 | 80 | 80 | 80 | 80 | 80 |
| Promotion of breastfeeding | 45,8 | 45,8 | 45,8 | 45,8 | 45,8 | 45,8 | 45,8 | 80 | 80 | 80 | 80 | 80 |
| Balanced energy supplementation | 0 | 0 | 0 | 0 | 0 | 0 | 0 | 80 | 80 | 80 | 80 | 80 |
| SAM - treatment for severe acute malnutrition | 14,4 | 14,4 | 14,4 | 14,4 | 14,4 | 14,4 | 14,4 | 80 | 80 | 80 | 80 | 80 |
| MAM - treatment for moderate acute malnutrition | 7,8 | 7,8 | 7,8 | 7,8 | 7,8 | 7,8 | 7,8 | 80 | 80 | 80 | 80 | 80 |

### Table K: Centre Global Stunting (<-2 SD) Rate

| AGE GROUP | BASELINE | | | | | | 80% COVERAGE SCENARIO | | | | | |
| --- | --- | --- | --- | --- | --- | --- | --- | --- | --- | --- | --- | --- |
|  | 2022 | 2023 | 2024 | 2025 | 2026 | 2027 | 2022 | 2023 | 2024 | 2025 | 2026 | 2027 |
| <1 month | 5,20 | 5,20 | 5,20 | 5,19 | 5,19 | 5,19 | 5,20 | 5,04 | 5,04 | 5,04 | 5,03 | 5,03 |
| 1-5 months | 5,20 | 5,20 | 5,20 | 5,20 | 5,20 | 5,19 | 5,20 | 5,11 | 5,11 | 5,11 | 5,11 | 5,10 |
| 6-11 months | 7,40 | 7,39 | 7,39 | 7,39 | 7,39 | 7,39 | 7,40 | 7,14 | 7,14 | 7,14 | 7,14 | 7,14 |
| 12-23 months | 12,90 | 12,90 | 12,90 | 12,90 | 12,90 | 12,90 | 12,90 | 11,45 | 11,26 | 11,26 | 11,26 | 11,26 |
| 24-59 months | 14,43 | 14,43 | 14,43 | 14,43 | 14,43 | 14,43 | 14,43 | 13,20 | 12,83 | 12,42 | 12,00 | 11,95 |
| Total (0-59 months) | 12,29 | 12,49 | 12,71 | 12,89 | 13,04 | 13,18 | 12,29 | 11,42 | 11,36 | 11,23 | 11,06 | 11,12 |

### Table L: Centre No. Of Stunting Cases Averted by Intervention (Total (0-59mo)) 2022-2027

| INTERVENTION | 80% COVERAGE SCENARIO | | | | | |
| --- | --- | --- | --- | --- | --- | --- |
|  | 2022 | 2023 | 2024 | 2025 | 2026 | 2027 |
| Calcium supplementation | 0 | 27 | 53 | 61 | 66 | 54 |
| Micronutrient supplementation (iron & MMN) | 0 | 9 | 17 | 20 | 21 | 18 |
| Balanced energy supplementation | 0 | 21 | 42 | 48 | 52 | 43 |
| Age-appropriate breastfeeding practices | 0 | 4 | 9 | 11 | 12 | 10 |
| Appropriate complementary feeding | 0 | 220 | 429 | 571 | 673 | 572 |
| Vitamin A supplementation | 0 | 173 | 233 | 273 | 295 | 250 |
| Zinc supplementation | 0 | 3 295 | 3 807 | 4 250 | 4 423 | 3 749 |

### Table M: Centre No. Of Additional Lives Saved by Intervention (Total (0-59mo)) 2022-2027

|  | 80% COVERAGE SCENARIO | | | | | |
| --- | --- | --- | --- | --- | --- | --- |
|  | 2022 | 2023 | 2024 | 2025 | 2026 | 2027 |
| Folic acid fortification | 0 | 9 | 7 | 6 | 5 | 3 |
| Calcium supplementation | 0 | 17 | 15 | 13 | 10 | 7 |
| Micronutrient supplementation (iron and multiple micronutrients) | 0 | 7 | 6 | 5 | 4 | 3 |
| Balanced energy supplementation | 0 | 20 | 17 | 14 | 11 | 8 |
| Age appropriate breastfeeding practices due to promotion | 0 | 15 | 12 | 10 | 9 | 6 |
| Vitamin A supplementation | 0 | 38 | 37 | 34 | 30 | 24 |
| Zinc supplementation | 0 | 162 | 163 | 154 | 139 | 115 |
| Complementary feeding via reduction in stunting | 0 | 4 | 6 | 6 | 6 | 5 |
| Complementary feeding via reduction in wasting | 0 | 7 | 7 | 6 | 5 | 4 |
| SAM - treatment for severe acute malnutrition | 0 | 59 | 56 | 50 | 43 | 35 |
| MAM - treatment for moderate acute malnutrition | 0 | 364 | 333 | 296 | 252 | 202 |

## SAHEL 80% COVERAGE SCENARIO

### Table N: Sahel Coverage Summary Key Intervention 2022-2027

| INTERVENTIONS | BASELINE | | | | | | 80% SCALE UP SCENARIO | | | | | |
| --- | --- | --- | --- | --- | --- | --- | --- | --- | --- | --- | --- | --- |
|  | 2022 | 2023 | 2024 | 2025 | 2026 | 2027 | 2022 | 2023 | 2024 | 2025 | 2026 | 2027 |
| Folic acid fortification | 14,5 | 14,5 | 14,5 | 14,5 | 14,5 | 14,5 | 14,5 | 80 | 80 | 80 | 80 | 80 |
| Micronutrient supplementation (iron and multiple micronutrients) | 45,6 | 45,6 | 45,6 | 45,6 | 45,6 | 45,6 | 45,6 | 80 | 80 | 80 | 80 | 80 |
| *Iron supplementation in pregnancy* | *45,6* | *45,6* | *45,6* | *45,6* | *45,6* | *45,6* | *45,6* | *45,6* | *45,6* | *45,6* | *45,6* | *45,6* |
| *Multiple micronutrient supplementation in pregnancy* | *0,0* | *0,0* | *0,0* | *0,0* | *0,0* | *0,0* | *0,0* | *34,4* | *34,4* | *34,4* | *34,4* | *34,4* |
| Calcium supplementation | 0 | 0 | 0 | 0 | 0 | 0 | 0 | 80 | 80 | 80 | 80 | 80 |
| Complementary feeding - supplementary feeding and education | 28,9 | 28,9 | 28,9 | 28,9 | 28,9 | 28,9 | 28,9 | 80 | 80 | 80 | 80 | 80 |
| Vitamin A supplementation | 84,7 | 84,7 | 84,7 | 84,7 | 84,7 | 84,7 | 84,7 | 84,7 | 84,7 | 84,7 | 84,7 | 84,7 |
| Zinc supplementation | 0 | 0 | 0 | 0 | 0 | 0 | 0 | 80 | 80 | 80 | 80 | 80 |
| Promotion of breastfeeding | 61,1 | 61,1 | 61,1 | 61,1 | 61,1 | 61,1 | 61,1 | 80 | 80 | 80 | 80 | 80 |
| Balanced energy supplementation | 0 | 0 | 0 | 0 | 0 | 0 | 0 | 80 | 80 | 80 | 80 | 80 |
| SAM - treatment for severe acute malnutrition | 47,7 | 47,7 | 47,7 | 47,7 | 47,7 | 47,7 | 47,7 | 80 | 80 | 80 | 80 | 80 |
| MAM - treatment for moderate acute malnutrition | 20,3 | 20,3 | 20,3 | 20,3 | 20,3 | 20,3 | 20,3 | 80 | 80 | 80 | 80 | 80 |

### Table O: Sahel Global Stunting (<-2 SD) Rate

| AGE GROUP | BASELINE | | | | | | 80% COVERAGE SCENARIO | | | | | |
| --- | --- | --- | --- | --- | --- | --- | --- | --- | --- | --- | --- | --- |
|  | 2022 | 2023 | 2024 | 2025 | 2026 | 2027 | 2022 | 2023 | 2024 | 2025 | 2026 | 2027 |
| <1 month | 15,43 | 15,43 | 15,43 | 15,43 | 15,43 | 15,43 | 15,43 | 15,00 | 15,00 | 15,00 | 15,00 | 15,00 |
| 1-5 months | 15,43 | 15,43 | 15,43 | 15,43 | 15,43 | 15,43 | 15,43 | 15,13 | 15,13 | 15,13 | 15,13 | 15,13 |
| 6-11 months | 23,76 | 23,76 | 23,76 | 23,76 | 23,76 | 23,76 | 23,76 | 22,98 | 22,98 | 22,98 | 22,98 | 22,98 |
| 12-23 months | 44,62 | 44,62 | 44,62 | 44,62 | 44,62 | 44,62 | 44,62 | 41,29 | 40,78 | 40,78 | 40,78 | 40,78 |
| 24-59 months | 50,43 | 50,43 | 50,43 | 50,43 | 50,43 | 50,43 | 50,43 | 47,77 | 46,90 | 45,89 | 44,88 | 44,75 |
| Total (0-59 months) | 42,43 | 42,42 | 42,48 | 42,44 | 42,37 | 42,24 | 42,43 | 40,09 | 39,57 | 38,96 | 38,34 | 38,16 |

### Table P: Sahel No. Of Stunting Cases Averted by Intervention (Total (0-59mo))

| INTERVENTION | 80% COVERAGE SCENARIO | | | | | |
| --- | --- | --- | --- | --- | --- | --- |
|  | 2022 | 2023 | 2024 | 2025 | 2026 | 2027 |
| Calcium supplementation | 0 | 67 | 133 | 166 | 199 | 206 |
| Micronutrient supplementation (iron and MMN) | 0 | 57 | 113 | 141 | 169 | 175 |
| Balanced energy supplementation | 0 | 58 | 115 | 143 | 172 | 178 |
| Age-appropriate breastfeeding practices | 0 | 33 | 57 | 81 | 105 | 109 |
| Appropriate complementary feeding | 0 | 585 | 1 197 | 1 706 | 2 224 | 2 306 |
| Zinc supplementation | 0 | 6 329 | 7 542 | 8 979 | 10 419 | 10 618 |

### Table Q: Sahel No. Of Additional Lives Saved (Total (0-59mo)) 2022-2027

|  | 80% COVERAGE SCENARIO | | | | | |
| --- | --- | --- | --- | --- | --- | --- |
|  | 2022 | 2023 | 2024 | 2025 | 2026 | 2027 |
| Folic acid fortification | 0 | 8 | 8 | 8 | 9 | 9 |
| Calcium supplementation | 0 | 29 | 30 | 31 | 31 | 32 |
| Micronutrient supplementation (iron and multiple micronutrients) | 0 | 28 | 29 | 30 | 31 | 32 |
| Balanced energy supplementation | 0 | 32 | 34 | 35 | 35 | 36 |
| Age appropriate breastfeeding practices due to promotion | 0 | 24 | 25 | 25 | 26 | 27 |
| Zinc supplementation | 0 | 174 | 182 | 189 | 195 | 198 |
| Complementary feeding via reduction in stunting | 0 | 8 | 12 | 14 | 16 | 17 |
| Complementary feeding via reduction in wasting | 0 | 12 | 12 | 12 | 12 | 12 |
| SAM - treatment for severe acute malnutrition | 0 | 39 | 39 | 40 | 41 | 41 |
| MAM - treatment for moderate acute malnutrition | 0 | 510 | 510 | 519 | 529 | 538 |
