# Supplementary material for: Investing in human development and building state resilience in fragile contexts: A case study of early nutrition investments in Burkina Faso
Source: PLOS Glob Public Health. 2023 Mar 29;3(3):e0001737. doi: 10.1371/journal.pgph.0001737 (PMC10058088; doi:10.1371/journal.pgph.0001737)
Supplement: S4 Text — (DOCX) [file pgph.0001737.s004.docx]

# S4: Costing Assumptions

### Table A: All Default Staff Baseline Costs

| INTERVENTION STAFF | ANNUAL SALARY 2022 (XOF) | ANNUAL REAL SALARY INCREASE (%) | AVERAGE DAYS WORKED PER YEAR | HOURS IN A WORKDAY |
| --- | --- | --- | --- | --- |
| Generalists/primary care doctors | 3017085,078 | 3,1 | 240 | 6 |
| Ob\Gyns | 4528954,689 | 3,1 | 240 | 6 |
| Pediatricians | 4528954,689 | 3,1 | 240 | 6 |
| Other specialist doctors | 4528954,689 | 3,1 | 240 | 6 |
| Clinical officers/surgical technicians | 1863378,354 | 3,1 | 240 | 6 |
| Nurses | 1863378,354 | 3,1 | 240 | 6 |
| Midwives | 1863378,354 | 3,1 | 240 | 6 |
| Assistant nurses and midwives | 1411412,758 | 3,1 | 240 | 6 |
| Nursing aides | 1411412,758 | 3,1 | 240 | 6 |
| Laboratory technicians/assistants | 1863378,354 | 3,1 | 240 | 6 |
| Pharmaceutical technicians/ assistants | 1863378,354 | 3,1 | 240 | 6 |
| Radiographers/X-ray technicians | 1863378,354 | 3,1 | 240 | 6 |
| Emergency medical technicians | 1863378,354 | 3,1 | 240 | 6 |
| Community health workers | 1082411,219 | 3,1 | 240 | 6 |
| Other | 1411412,758 | 3,1 | 240 | 6 |

Staff baseline data is pre-populated with assumptions for salaries, benefits, and time utilization drawn from WHO CHOICE. These data points are used to estimate a cost per minute, which is combined with information from Treatment Inputs editors to estimate the labor costs for delivering the various interventions. Users are encouraged to revise these assumptions if more accurate local information is available.

Salaries are assumed to be an annual salary for a full-time staff person, denoted in local currency or US dollars, depending on the user’s selection in LiST Configuration. Benefits are calculated as a percentage of those salary costs, and entries for days per year and time worked per day are used to convert the assumptions for what a full-time staff person can do into a cost per minute.

### Table B: Target Population

| INTERVENTION | TARGET POPULATION | SOURCE |
| --- | --- | --- |
| Folic acid supplementation/fortification | Women of reproductive age (15-49) | Custom |
| Calcium supplementation | Pregnant women | Default |
| Iron supplementation in pregnancy |  | Default |
| Multiple micronutrient supplementation in pregnancy |  | Default |
| Balanced energy supplementation |  | Default |
| Promotion of breastfeeding | Live births | Default |
| Complementary feeding - supplementary feeding and education | Children 6-23 months | Custom |
| Vitamin A supplementation |  | Custom |
| Zinc supplementation |  | Custom |
| SAM - treatment for severe acute malnutrition |  | Custom |
| MAM - treatment for moderate acute malnutrition |  | Custom |

## POPULATION IN NEED (DISEASE INCIDENCE) %

Population in need is determined by incidence and prevalence of conditions, as well as by treatment guidelines.

Default values automatically adjusted by LiST per regional model

| **Table C: National** **Population in Need** | | | | | | |
| --- | --- | --- | --- | --- | --- | --- |
| INTERVENTION | 2022 | 2023 | 2024 | 2025 | 2026 | 2027 |
| Folic acid supplementation/fortification | 100 | 100 | 100 | 100 | 100 | 100 |
| Calcium supplementation | 100 | 100 | 100 | 100 | 100 | 100 |
| Micronutrient supplementation (iron and multiple micronutrients) | 100 | 100 | 100 | 100 | 100 | 100 |
| Balanced energy supplementation | 3,9 | 3,9 | 3,9 | 3,9 | 3,9 | 3,9 |
| Promotion of breastfeeding | 100 | 100 | 100 | 100 | 100 | 100 |
| Complementary feeding - supplementary feeding and education* | 43,7 | 43,7 | 43,7 | 43,7 | 43,7 | 43,7 |
| Vitamin A supplementation | 100 | 100 | 100 | 100 | 100 | 100 |
| Zinc supplementation | 100 | 100 | 100 | 100 | 100 | 100 |
| SAM - treatment for severe acute malnutrition | 3,1 | 1,3 | 1,3 | 1,3 | 1,3 | 1,3 |
| MAM - treatment for moderate acute malnutrition | 10,7 | 10,7 | 10,7 | 10,7 | 10,7 | 10,7 |

| **Table D: Nord Population in Need** | | | | | | |
| --- | --- | --- | --- | --- | --- | --- |
| INTERVENTION | **2022** | **2023** | **2024** | **2025** | **2026** | **2027** |
| Folic acid supplementation/fortification | 100 | 100 | 100 | 100 | 100 | 100 |
| Calcium supplementation | 100 | 100 | 100 | 100 | 100 | 100 |
| Micronutrient supplementation (iron and multiple micronutrients) | 100 | 100 | 100 | 100 | 100 | 100 |
| Balanced energy supplementation | 6,4 | 6,4 | 6,4 | 6,4 | 6,4 | 6,4 |
| Promotion of breastfeeding | 100 | 100 | 100 | 100 | 100 | 100 |
| Complementary feeding - supplementary feeding and education | 43,7 | 43,7 | 43,7 | 43,7 | 43,7 | 43,7 |
| Vitamin A supplementation | 100 | 100 | 100 | 100 | 100 | 100 |
| Zinc supplementation | 100 | 100 | 100 | 100 | 100 | 100 |
| SAM - treatment for severe acute malnutrition | 2,8 | 1,5 | 1,5 | 1,5 | 1,5 | 1,5 |
| MAM - treatment for moderate acute malnutrition | 12,7 | 12,7 | 12,7 | 12,7 | 12,7 | 12,7 |

| **Table E: Centre Population in Need** | | | | | | |
| --- | --- | --- | --- | --- | --- | --- |
| INTERVENTION | 2022 | 2023 | 2024 | 2025 | 2026 | 2027 |
| Folic acid supplementation/fortification | 100 | 100 | 100 | 100 | 100 | 100 |
| Calcium supplementation | 100 | 100 | 100 | 100 | 100 | 100 |
| Micronutrient supplementation (iron and multiple micronutrients) | 100 | 100 | 100 | 100 | 100 | 100 |
| Balanced energy supplementation | 6,4 | 6,4 | 6,4 | 6,4 | 6,4 | 6,4 |
| Promotion of breastfeeding | 100 | 100 | 100 | 100 | 100 | 100 |
| Complementary feeding - supplementary feeding and education | 43,7 | 43,7 | 43,7 | 43,7 | 43,7 | 43,7 |
| Vitamin A supplementation | 100 | 100 | 100 | 100 | 100 | 100 |
| Zinc supplementation | 100 | 100 | 100 | 100 | 100 | 100 |
| SAM - treatment for severe acute malnutrition | 3,2 | 1,2 | 1,1 | 1,1 | 1,1 | 1,1 |
| MAM - treatment for moderate acute malnutrition | 9,3 | 9,3 | 9,3 | 9,3 | 9,3 | 9,3 |

| **Table F: Sahel Population in Need** | | | | | | |
| --- | --- | --- | --- | --- | --- | --- |
| INTERVENTION | 2022 | 2023 | 2024 | 2025 | 2026 | 2027 |
| Folic acid supplementation/fortification | 100 | 100 | 100 | 100 | 100 | 100 |
| Calcium supplementation | 100 | 100 | 100 | 100 | 100 | 100 |
| Micronutrient supplementation (iron and multiple micronutrients) | 100 | 100 | 100 | 100 | 100 | 100 |
| Balanced energy supplementation | 6,4 | 6,4 | 6,4 | 6,4 | 6,4 | 6,4 |
| Promotion of breastfeeding | 100 | 100 | 100 | 100 | 100 | 100 |
| Complementary feeding - supplementary feeding and education | 43,7 | 43,7 | 43,7 | 43,7 | 43,7 | 43,7 |
| Vitamin A supplementation | 100 | 100 | 100 | 100 | 100 | 100 |
| Zinc supplementation | 100 | 100 | 100 | 100 | 100 | 100 |
| SAM - treatment for severe acute malnutrition | 5,8 | 2,2 | 2,1 | 2,1 | 2,1 | 2,1 |
| MAM - treatment for moderate acute malnutrition | 18,4 | 18,4 | 18,4 | 18,4 | 18,4 | 18,4 |

### Treatment Inputs

Default LiST values used.

### Other-Recurrent and Capital Costs

Default LiST values used.

### Delivery Channels

Default LiST values used.

### Program Costs

Default LiST values used.

### Logistics and Wastage

Default LiST values used.

### Infrastructure Investment

Default LiST values used.

### Other Health System Costs

Default LiST values used.

### Inefficiencies

Default LiST values used.

## CALCULATION OF NO OF VISITS

### Table G: Breakdown of Delivery Channels

| INTERVENTION | LIST: DELIVERY CHANNEL | | | |
| --- | --- | --- | --- | --- |
|  | **COMMUNITY** | **OUTREACH** | **CLINIC** | **HOSPITAL** |
| Folic acid fortification/ supp | 100% | - | - | - |
| Balanced energy supplementation, | 50% | 50% | - | - |
| Micronutrient supplementation | - | - | 100% |  |
| iron supplementation | 50% | - | 25% | 25% |
| Calcium supplementation | - | - | 100% | - |
| Vitamin A supplementation | 50% |  | 50% | - |
| Zinc supplementation | 50% | 50% | - | - |
| Complementary feeding - supplementary feeding and education | 100% | - | - | - |
| Promotion of breastfeeding | 40% | 10% | 50% |  |
| SAM - treatment for severe acute malnutrition | - | - | - | 80% |
| MAM - treatment for moderate acute malnutrition | 50% | - | 50% | - |

### Table H: National Intervention Services Delivered in Clinic/ Hospital

|  | | LIST TOTAL NO SERVICES BY DELIVERY CHANNEL 2023 | | | | |
| --- | --- | --- | --- | --- | --- | --- |
| TARGET POPULATION | **INTERVENTION** | **CLINIC** | | **HOSPITAL** | | **TOTAL** |
| Pregnancy | Micronutrient supplementation | 134734 | | - | | Not included |
|  | Iron supplementation | 180180 | | 180180 | | Not included |
|  | Calcium supplementation | 855455 | | - | | 855455 |
| Children 6-23 months | Vitamin A supplementation | 448131 | | - | | 448131 |
| Live births | Promotion of breastfeeding | 317850 | | - | | 317850 |
| Children with SAM | Treatment for SAM |  | | 9013 | | 9013 |
| Children with MAM | Treatment for MAM | 48591 | | - | | 48591 |
| Proportion (%) 10kms + away from health facilities, 2020* | | | 19,50% | | 1679040 | |
| Cost for 5 USD travel vouchers | | |  | | 1637064,00 | |

*Annuaire Statistique 2020, Ministère de la Santé Burkina Faso

### Table I: Nord Intervention Services Delivered in Clinic/ Hospital

|  | | LIST TOTAL NO SERVICES BY DELIVERY CHANNEL 2023 | | | | |
| --- | --- | --- | --- | --- | --- | --- |
| TARGET POPULATION | **INTERVENTION** | **CLINIC** | | **HOSPITAL** | **TOTAL** | |
| Pregnancy | Micronutrient supplementation | 6628 | | - | Not included | |
|  | Iron supplementation | 17553 | | 17553 | Not included | |
|  | Calcium supplementation | 76841 | | - | 76841 | |
| Children 6-23 months | Vitamin A supplementation, | 50398 | | - | 50398 | |
| Live births | Promotion of breastfeeding | 31980 | | - | 31980 | |
| Children with SAM | Treatment for SAM |  | | 1021 | 1021 | |
| Children with MAM | Treatment for MAM | 5395 | | - | 5395 | |
| Proportion (%) 10kms + away from health facilities, 2020* | | | 14,50% | | | 165635 |
| Cost for 5 US$ travel vouchers | | | 120085,38 | | | |

*Annuaire Statistique 2020, Ministère de la Santé Burkina Faso

### Table J: Centre Intervention Services Delivered in Clinic/ Hospital

|  | | LIST TOTAL NO SERVICES BY DELIVERY CHANNEL 2023 | | | | | |
| --- | --- | --- | --- | --- | --- | --- | --- |
| TARGET POPULATION | **INTERVENTION** | **CLINIC** | | **HOSPITAL** | | | **TOTAL** |
| Pregnancy | Micronutrient supplementation | 14046 | | - | Not included | | |
|  | Iron supplementation | 16412 | | 16412 | Not included | | |
|  | Calcium supplementation | 79695 | | - | 79695 | | |
| Children 6-23 months | Vitamin A supplementation | 44269 | | - | 44269 | | |
| Live births | Promotion of breastfeeding | 27456 | | - | 27456 | | |
| Children with SAM | Treatment for SAM |  | | 851 | 851 | | |
| Children with MAM | Treatment for MAM | 4135 | | - | 4135 | | |
| Proportion (%) 10kms + away from health facilities, 2020* | | | 3,90% | | | 156406 | |
| Cost for 5 US$ travel vouchers | | | 30499,17 | | | | |

*Annuaire Statistique 2020, Ministère de la Santé Burkina Faso

### Table K: Sahel Intervention Services Delivered in Clinic/ Hospital

|  | | LIST TOTAL NO SERVICES BY DELIVERY CHANNEL 2023 | | | | | |
| --- | --- | --- | --- | --- | --- | --- | --- |
| TARGET POPULATION | **INTERVENTION** | **CLINIC** | | **HOSPITAL** | | | **TOTAL** |
| Pregnancy | Micronutrient supplementation | 29786 | | - | Not included | | |
|  | Iron supplementation | 9871 | | 9871 | Not included | | |
|  | Calcium supplementation | 69270 | | - | | | 69270 |
| Children 6-23 months | Vitamin A supplementation | 39005 | | - | | | 39005 |
| Live births | Promotion of breastfeeding | 26685 | | - | | | 26685 |
| Children with SAM | Treatment for SAM | - | | 1343 | | | 1343 |
| Children with MAM | Treatment for MAM | 7162 | | - | | | 7162 |
| Proportion (%) 10kms + away from health facilities, 2020* | | | 44% | | | 143465 | |
| Cost for 5 US$ travel vouchers | | | 315623 | | | | |

*Annuaire Statistique 2020, Ministère de la Santé Burkina Faso

## COST PER INTERVENTION 2022-2027

### Table L: National Total Intervention Costs 2022-2027 US$

| INTERVENTIONS | 2022 | 2023 | 2024 | 2025 | 2026 | 2027 |
| --- | --- | --- | --- | --- | --- | --- |
| Folic acid fortification | 26238 | 149466 | 154288 | 159205 | 164196 | 169249 |
| Calcium supplementation in pregnancy | 0 | 9500329 | 9648116 | 9792102 | 9950278 | 10107545 |
| Iron supplementation in pregnancy | 614805 | 632463 | 648682 | 665027 | 682740 | 700817 |
| Multiple micronutrient supplementation in pregnancy | 0 | 526216 | 535242 | 544107 | 553813 | 563525 |
| Balanced energy supplementation | 0 | 2190479 | 2222982 | 2254514 | 2289215 | 2323601 |
| Breastfeeding promotion | 854120 | 2357286 | 2431762 | 2507788 | 2590613 | 2676205 |
| Complementary feeding - supplementary feeding and education | 5686907 | 15860181 | 16187659 | 16443005 | 16706284 | 16990703 |
| Vitamin A Supplementation | 600148 | 613989 | 629878 | 643156 | 656934 | 671742 |
| Zinc supplementation | 0 | 5733526 | 5848033 | 5936245 | 6027094 | 6125328 |
| Treatment for severe acute malnutrition (SAM) | 1892861 | 1377717 | 1414072 | 1442310 | 1472783 | 1506759 |
| Treatment for moderate acute malnutrition (MAM) | 1105391 | 4416929 | 4496700 | 4555737 | 4616301 | 4681995 |

### Table M: Nord Total Intervention Costs 2022-2027 US$

| INTERVENTIONS | 2022 | 2023 | 2024 | 2025 | 2026 | 2027 |
| --- | --- | --- | --- | --- | --- | --- |
| Folic acid fortification | 2 203 | 12 546 | 12 951 | 13 363 | 13 782 | 14 205 |
| Calcium supplementation in pregnancy | 0 | 853369 | 873760 | 894323 | 915906 | 937874 |
| Iron supplementation in pregnancy | 59325 | 61616 | 63715 | 65874 | 68160 | 70528 |
| Multiple micronutrient supplementation in pregnancy | 0 | 25885 | 26545 | 27213 | 27916 | 28635 |
| Balanced energy supplementation | 0 | 322206 | 329672 | 337184 | 345063 | 353066 |
| Breastfeeding promotion | 226969 | 237175 | 246584 | 256344 | 266781 | 277690 |
| Complementary feeding - supplementary feeding and education | 390404 | 1508389 | 1473718 | 1508808 | 1545010 | 1583094 |
| Vitamin A Supplementation | 66167 | 69051 | 67809 | 69786 | 71841 | 74011 |
| Zinc supplementation | 0 | 545289 | 532403 | 544709 | 557390 | 570722 |
| Treatment for severe acute malnutrition | 131047 | 156046 | 151166 | 155546 | 160317 | 166141 |
| Treatment for moderate acute malnutrition (MAM) | 261577 | 490390 | 477903 | 488008 | 498381 | 509263 |

### Table N: Centre Total Intervention Costs 2022-2027 US$

| INTERVENTIONS | 2022 | 2023 | 2024 | 2025 | 2026 | 2027 |
| --- | --- | --- | --- | --- | --- | --- |
| Folic acid supplementation or fortification | 3881 | 22105 | 22818 | 23544 | 24282 | 25028 |
| Calcium supplementation in pregnancy | 0 | 885061 | 760602 | 628969 | 505548 | 375519 |
| Iron supplementation in pregnancy | 64543 | 57610 | 50000 | 41766 | 33916 | 25458 |
| Multiple micronutrient supplementation in pregnancy | 0 | 54859 | 47219 | 39110 | 31488 | 23429 |
| Balanced energy supplementation | 0 | 334172 | 286977 | 237139 | 190462 | 141366 |
| Breastfeeding promotion | 129697 | 203626 | 178339 | 149730 | 121334 | 89379 |
| Complementary feeding - supplementary feeding and education | 897993 | 1566760 | 1326017 | 1129082 | 923606 | 717276 |
| Vitamin A Supplementation | 50671 | 60653 | 51597 | 44163 | 36319 | 28358 |
| Zinc supplementation | 0 | 566391 | 479044 | 407621 | 333207 | 258585 |
| Treatment for severe acute malnutrition (SAM) | 62676 | 130144 | 105513 | 87280 | 69735 | 53312 |
| Treatment for moderate acute malnutrition (MAM) | 37034 | 375848 | 317290 | 269464 | 219836 | 170256 |

### Table O: Sahel Total Intervention Costs 2022-2027 US$

| INTERVENTIONS | 2022 | 2023 | 2024 | 2025 | 2026 | 2027 |
| --- | --- | --- | --- | --- | --- | --- |
| Folic acid supplementation or fortification | 1 675 | 9 544 | 9 852 | 10 165 | 10 484 | 10 806 |
| Calcium supplementation in pregnancy | 0 | 769287 | 791094 | 813365 | 836481 | 860172 |
| Iron supplementation in pregnancy | 33017 | 34649 | 35985 | 37373 | 38831 | 40351 |
| Multiple micronutrient supplementation in pregnancy | 0 | 116333 | 119818 | 123390 | 127108 | 130930 |
| Balanced energy supplementation | 0 | 290459 | 298481 | 306661 | 315140 | 323815 |
| Breastfeeding promotion | 143005 | 197902 | 206704 | 215911 | 225689 | 235952 |
| Complementary feeding - supplementary feeding and education | 483128 | 1380433 | 1344912 | 1383370 | 1423138 | 1464631 |
| Vitamin A Supplementation | 54541 | 53440 | 52332 | 54110 | 55961 | 57905 |
| Zinc supplementation | 0 | 499033 | 485870 | 499424 | 513423 | 528015 |
| Treatment for severe acute malnutrition (SAM) | 317076 | 205344 | 198503 | 205286 | 212690 | 221429 |
| Treatment for moderate acute malnutrition (MAM) | 160681 | 651045 | 632705 | 649103 | 665977 | 683513 |
