# Supplementary material for: Investing in human development and building state resilience in fragile contexts: A case study of early nutrition investments in Burkina Faso
Source: PLOS Glob Public Health. 2023 Mar 29;3(3):e0001737. doi: 10.1371/journal.pgph.0001737 (PMC10058088; doi:10.1371/journal.pgph.0001737)
Supplement: S5 Text — (DOCX) [file pgph.0001737.s005.docx]

# S5: Productivity Estimates

### Table A: Assumptions for Productivity Estimates

| VARIABLES | NATIONAL | NORD | CENTRE | SAHEL | ASSUMPTION RATIONALE | SOURCE |
| --- | --- | --- | --- | --- | --- | --- |
| Cohort size | 782000 | 70000 | 78000 | 64000 | Single year birth cohort nationally and by region from LiST models rounded to nearest 1,000 | List Default |
| Grade 1 enrolment rate | 1 | 1 | 1 | 0.73 | Gross enrolment by region  to a maximum of 1. | Direction Generale Des Etudes Et Des Statistiques Sectorielles (DGESS)/ Ministere De L’education Nationale (MENA). 2017. *Annuaire Statistique De L’enseignement Primaire 2016/2027.* Ouagadougou, Burkina Faso: DGESS/MENA. Via <https://burkinafaso.opendataforafrica.org/gsykdac/evolution-du-taux-brut-d-admission-au-cp1-par-r%C3%A9gion-en> |
| Impact of nutrition interventions on z-score | 0.0912 | 0.0906 | 0.0859 | 0.0946 | Stunting rates from national and regional LiST models. Rates converted to improvements in z-score assuming a normal distribution. |  |
| Impact of 1 SD increase in z-score on years of schooling | 0.48 |  |  |  | Following Fink et al (2016) and Desmond et al (2021) we assume the improvements to schooling apply to all those who see an improved z-score. The rate of improved schooling is based on Adair et al’s (2013) analysis of 5 birth cohorts in LMICs. | Fink, G., Peet, E., Danaei, G., Andrews, K., McCoy, D. C., Sudfeld, C. R., Smith Fawzi, M. C., Ezzati, M., & Fawzi, W. W. (2016). Schooling and wage income losses due to early-childhood growth faltering in developing countries: National, regional, and global estimates. *American Journal of Clinical Nutrition*, *104*(1), 104–112. <https://doi.org/10.3945/ajcn.115.123968>  Adair, L. S., Fall, C. H., Osmond, C., Stein, A. D., Martorell, R., Ramirez-Zea, M., Sachdev, H S., Dahly, D L., Bas, I., Norris, S.A., Micklesfield, L., Hallal, P., & Victora, C.G. (2013). Associations of linear growth and relative weight gain during early life with adult health and human capital in countries of low and middle income: findings from five birth cohort studies. The Lancet, 382(9891), 525–534. <https://doi.org/10.1016/S0140-6736(13)60103-8>  Desmond, C., Erzse, A., Watt, K., Ward, K., Newell, M.-L., Hofman, K., & group, Inp. (2021). Realising the potential human development returns to investing in early and maternal nutrition: The importance of identifying and addressing constraints over the life course. PLOS Global Public Health, 1(10), e0000021. https://doi.org/10.1371/journal.pgph.0000021 |
| Impact of cash transfers on school drop-out | 0.3 |  |  |  | We assumed an impact on drop out which would lead to a maximum increase in enrolment of the intervention scenario at the national level no higher than the increase observed in a RCT of a conditional cash transfer in BF. | Akresh, R., De Walque, D., & Kazianga, H. (2013). Cash transfers and child schooling: evidence from a randomized evaluation of the role of conditionality (No. 6340; World Bank Policy Research Working Paper). |
| Baseline rate of primary school drop out | 10.8 | 13.5 | 5.0 | 23.7 | National and regional averages for primary school. | Direction Generale Des Etudes Et Des Statistiques Sectorielles (DGESS)/ Ministere De L’education Nationale (MENA). 2020. *Annuaire Statistique De L’enseignement Primaire 2019/2020.* Ouagadougou, Burkina Faso: DGESS/MENA. |
| Baseline rate of secondary school drop out | 7.2 | 11.0 | 1.0 | 31.8 | National and regional averages for secondary school. | Direction Generale Des Etudes Et Des Statistiques Sectorielles (DGESS)/ Ministere De L’education Nationale (MENA). 2020. *Annuaire Statistique De L’enseignement Primaire 2019/2020.* Ouagadougou, Burkina Faso: DGESS/MENA. |
| Baseline income (no education) | 35664 |  |  |  | Current minimum | International Labour Office (ILO). 2020. Global Wage Report 2020-21: Wages and minimum wages in the time of COVID-19. Geneva, Switzerland: ILO. |
| Returns to education | 10.5% |  |  |  | Sub-Saharan regional average of private returns | Patrinos, H. A., & Psacharopoulos, G. (2020). Chapter 4 - Returns to education in developing countries (S. Bradley & C. B. T.-T. E. of E. (Second E. Green (eds.); pp. 53–64). Academic Press. https://doi.org/https://doi.org/10.1016/B978-0-12-815391-8.00004-5 |
| Labour force participation rate | 0.7 |  |  |  |  | INSD, Enquête burkinabé sur les conditions de vie des ménages 2003 et enquête annuelle sur les conditions de vie des ménages (EA – QUIBB) 2005 via https://burkinafaso.opendataforafrica.org/ |
| Annual increase in real incomes | 2.7% |  |  |  | Average increase in GDP per capita 2015-19 | World Bank Group. “GDP per Capita (current US$)” *World Bank Development Indicators*. The World Bank Group. https://data.worldbank.org/indicator/NY.GDP.PCAP.CD |
| Years in the labour force | 40 |  |  |  | Assumption | Assumption |
| Cost of cash transfers | USD173 |  |  |  | Present value of USD20 per year for 80% of cohort from 7 years to 18 years. USD 20 per year cost of conditional cash transfer trial in BF inclusive of overhead. | Akresh, R., De Walque, D., & Kazianga, H. (2013). Cash transfers and child schooling: evidence from a randomized evaluation of the role of conditionality (No. 6340; World Bank Policy Research Working Paper). |
| Discount rate | 3% |  |  |  | Assumption | Assumption |
